# CircNUP50 is a novel therapeutic target that promotes cisplatin resistance in ovarian cancer by modulating p53 ubiquitination

**DOI:** 10.1186/s12951-024-02295-w

**Published:** 2024-01-19

**Authors:** Yunshu Zhu, Leilei Liang, Yuxi Zhao, Jian Li, Jia Zeng, Yihang Yuan, Ning Li, Lingying Wu

**Affiliations:** 1 Department of Gynecologic Oncology, National Cancer Center/National Clinical Research Center for Cancer/Cancer Hospital, Chinese Academy of Medical Sciences and Peking Union Medical College, Beijing, 100021 China; 2 Department of General Surgery, Tongren Hospital, Shanghai Jiao Tong University School of Medicine, 1111 XianXia Road, Shanghai, 200336 China

**Keywords:** Ovarian cancer, Platinum resistance, circRNA NUP50, p53, Nanodrug delivery

## Abstract

**Background:**

Most patients with ovarian cancer (OC) treated with platinum-based chemotherapy have a dismal prognosis owing to drug resistance. However, the regulatory mechanisms of circular RNA (circRNA) and p53 ubiquitination are unknown in platinum-resistant OC. We aimed to identify circRNAs associated with platinum-resistant OC to develop a novel treatment strategy.

**Methods:**

Platinum-resistant circRNAs were screened through circRNA sequencing and validated using quantitative reverse-transcription PCR in OC cells and tissues. The characteristics of circNUP50 were analysed using Sanger sequencing, oligo (dT) primers, ribonuclease R and fluorescence in situ hybridisation assays. Functional experimental studies were performed in vitro and in vivo. The mechanism underlying circNUP50-mediated P53 ubiquitination was investigated through circRNA pull-down analysis and mass spectrometry, luciferase reporters, RNA binding protein immunoprecipitation, immunofluorescence assays, cycloheximide chase assays, and ubiquitination experiments. Finally, a platinum and si-circNUP50 co-delivery nanosystem (Psc@DPP) was constructed to treat platinum-resistant OC in an orthotopic animal model.

**Results:**

We found that circNUP50 contributes to platinum-resistant conditions in OC by promoting cell proliferation, affecting the cell cycle, and reducing apoptosis. The si-circNUP50 mRNA sequencing and circRNA pull-down analysis showed that circNUP50 mediates platinum resistance in OC by binding p53 and UBE2T, accelerating p53 ubiquitination. By contrast, miRNA sequencing and circRNA pull-down experiments indicated that circNUP50 could serve as a sponge for miR-197-3p, thereby upregulating G3BP1 to mediate p53 ubiquitination, promoting OC platinum resistance. Psc@DPP effectively overcame platinum resistance in an OC tumour model and provided a novel idea for treating platinum-resistant OC using si-circNUP50.

**Conclusions:**

This study reveals a novel molecular mechanism by which circNUP50 mediates platinum resistance in OC by modulating p53 ubiquitination and provides new insights for developing effective therapeutic strategies for platinum resistance in OC.

**Graphical Abstract:**

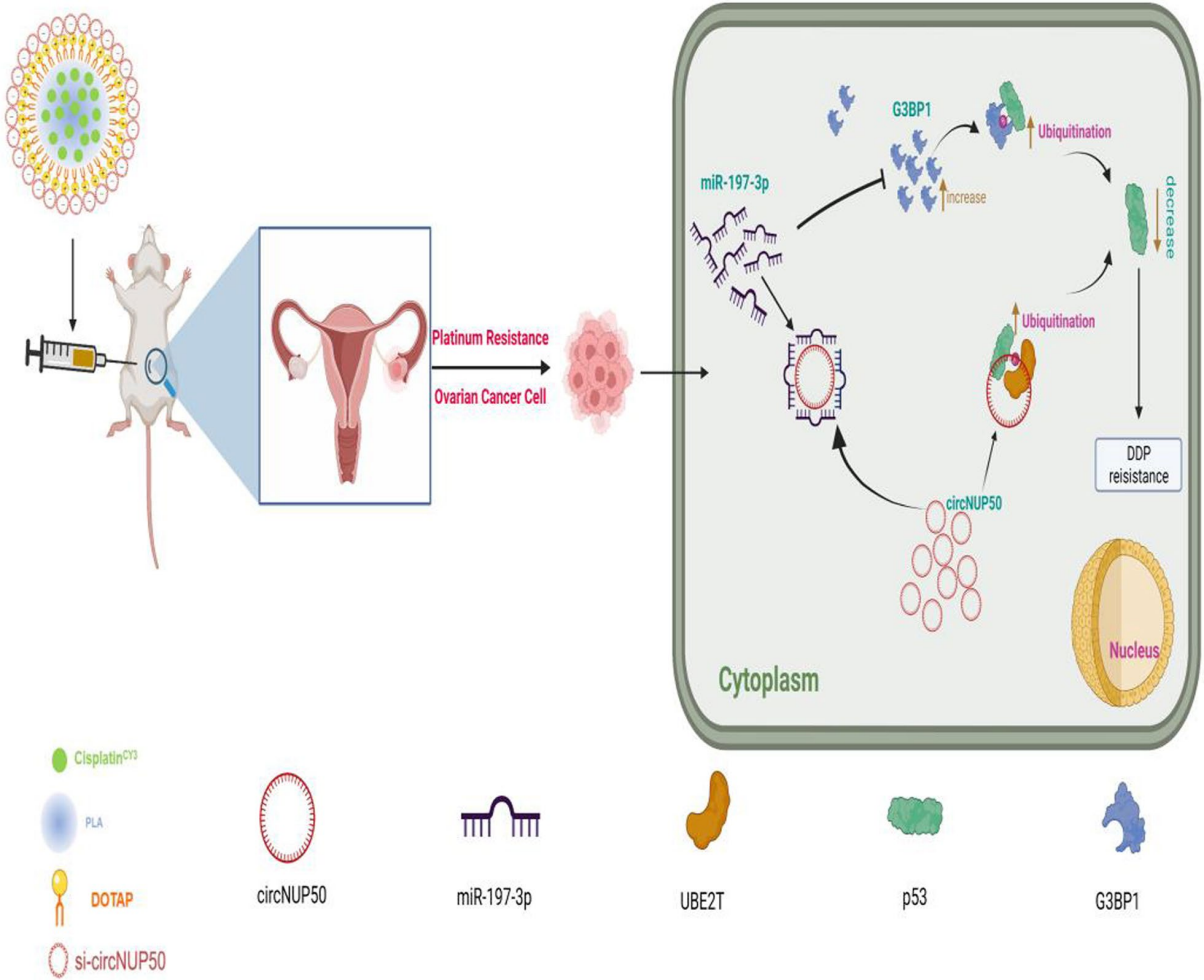

**Supplementary Information:**

The online version contains supplementary material available at 10.1186/s12951-024-02295-w.

## Introduction

Ovarian cancer (OC) is the most lethal gynaecological malignancy. Over 80% of the patients with epithelial OC present with advanced disease at the initial diagnosis [1], and the 5-year survival rate is only 41% [2]. The current recommended standard of care for first-line treatment is debulking surgery and platinum-based chemotherapy (with or without bevacizumab). Despite a high response after patients receive platinum-based chemotherapy, most patients with OC will relapse after completing first-line therapy [3] or succumb to cisplatin (DDP)-resistant disease with a median survival of less than 12 months [4]. Furthermore, efforts to overcome platinum resistance have been largely ineffective.

Circular RNAs (circRNAs) are a subtype of noncoding RNAs, a class of single-stranded noncoding RNAs with a covalently closed structure that lacks a 5′ terminal cap and a 3′ terminal poly(A) tail [5], making them more resistant to nucleic acid exonucleases and allowing them to regulate gene expression at the posttranscriptional level [6]. Owing to their conserved nature, abundance, and tissue specificity, circRNAs play an essential role in cancer progression and various cell biological and pathological processes, suggesting that these molecules have specific biological functions and the potential to serve as diagnostic markers and therapeutic targets. For example, the circRNA ITCH can inhibit the proliferation and promote the apoptosis of human OC cells by sponging miR-10a [7]. The circRNA CDR1as was downregulated in DDP-resistant tissues and cells, and circRNA CDR1as upregulation increased OC sensitivity to DDP, inhibiting proliferation and invasion and promoting apoptosis [8]. In addition, circ_0067934 increased carcinogenesis and DDP resistance in OC by reducing JNK phosphorylation through the microRNA-545-3p/PPA1 axis [9]. Furthermore, circITGB6 overexpression promotes M2 macrophage-dependent DDP resistance in OC in vivo and in vitro [10]. These findings show that circRNA is a unique biomarker for platinum-based treatment resistance and exerts a significant regulatory influence on developing platinum resistance in OC. However, no relevant studies have described the mechanism by which circRNAs regulate platinum resistance in OC or the impact of circRNA interactions with proteins; thus, limitations exist for developing target drugs for clinical translational applications.

The p53 protein is a key transcription factor that regulates cellular homeostasis. Phosphorylation of p53 at serine 15 (Ser15) and serine 20 (Ser20) is a key factor mediating DDP resistance in OC [11]. RING1A causes lysine 119 monoubiquitination of phosphorylated H2AX at the platinum DNA damage site to modify the susceptibility of OC cells to platinum treatment [12]. Furthermore, the expression of indoleamine 2,3-dioxygenase 1 (IDO1) was higher in platinum-resistant patients than in platinum-sensitive patients, and IDO1 could regulate the sensitivity of epithelial OC cells to DDP through reactive oxygen species/p53-dependent apoptosis [13]. In addition, HIF-1α and HDAC4 may mediate the crosstalk of p53 with RAS signalling to control DDP resistance in OC through apoptosis and autophagy dysregulation [14]. However, the mechanism by which circRNAs regulate p53 in platinum-resistant OC has not been fully investigated.

The main objectives of the present study were to identify circRNAs that play a role in mediation of platinum resistance in OC and how such circRNA crosstalk with p53 to establish resistance, thereby developing a nanodrug that can be clinically translated.

## Methods

### Patients and tissue samples

The sensitivity of patients with OC to platinum was evaluated by two experienced clinicians (all patients were evaluated for first-line treatment efficacy). Advanced OC tissue specimens were collected before the patients received their initial treatment and were not affected by treatment modalities such as chemotherapeutic drugs. Enrolled patients had no other comorbidities and no prior cancer. Fourteen platinum-resistant (defined as disease progression within 6 months after receiving platinum-based chemotherapy) and 25 platinum-sensitive (PS, defined as disease progression or relapse ≥ 6 months after the last dose of platinum-based therapy) freshly frozen OC tissues were obtained from the Cancer Hospital of the Chinese Academy of Medical Sciences between May 2016 and February 2022. Four OC platinum-resistant tissues and five OC PS tissues were used for circRNA microarray analysis. The remaining 10 OC platinum-resistant and 20 OC PS tissues were used for validation using quantitative reverse-transcription PCR (qRT-PCR). GENESEED (Guangzhou, China) performed circRNA-seq to examine the dysregulated circRNA expression in the four platinum-resistant OC tissues and five PS OC tissues. Clinical information of the patients is shown in Additional file 1: Table S1. The study was approved by the Ethics Committee of the Cancer Hospital of the Chinese Academy of Medical Sciences (NCC2021C-120).

### OC cell lines

OC cell lines (SKOV3 and A2780) were purchased from the Chinese Academy of Sciences Cell Bank (Shanghai, China). SKOV3 (the IC_50_ for DDP is 5.1 μM) and SKOV3/DDP (the IC_50_ for DDP is 52.7 μM) cells were cultured in Dulbecco's Modified Eagle Medium (Gibco, Thermo Fisher Scientific, Waltham, MA, USA) supplemented with 10% foetal bovine serum (FBS; Invitrogen, Carlsbad, CA, USA) supplemented with 1% penicillin and streptomycin. A2780 (the IC_50_ for DDP is 4.8 μM) and A2780/DDP (the IC_50_ for DDP is 42.4 μM) cells were cultured in RPMI-1640 (Gibco) containing 10% FBS and 1% penicillin and streptomycin. DDP (6 μM) was added to the medium used for culturing SKOV3/DDP and A2780/DDP cells to maintain resistance. All cells were cultured in a humidified incubator at 37 °C in an atmosphere of 5% CO_2_.

### CircNUP50 transfection

GenePharma (Shanghai, China) synthesised small interfering RNAs (siRNAs) that target the junction region of the circNUP50 sequence. GeneChem Company (Shanghai, China) created lentiviral shRNA plasmids and packaged them in pMD2. The GandpsPAX2 vector (Addgene, Cambridge, MA, USA) was used to construct plasmids for transfection into cells to generate the circNUP50 knockdown stable transfer cell lines sh-circNUP50-SKOV3/DDP and sh-circNUP50-A2780/DDP. These siRNAs were transfected into SKOV3/DDP and A2780/DDP cells using siRNA-Mate (GenePharma). The cells were used for the following assays 48 h after infection. The full-length siRNA and shRNA sequences are presented in Additional file 1: Table S2.

### qRT–PCR

The RNA-easy Isolation Reagent (No. RC112-01, Vazyme, China) was used to extract total RNA. The qRT-PCR experiment was conducted using a HiScript III 1st Strand cDNA Synthesis Kit (No. R312-01, Vazyme) and ChamQ™ Universal SYBR^®^ qPCR Master Mix (No. Q712-02, Vazyme) according to the manufacturer’s instruction. Additional file 1: Table S3 contains information on the sequences. The circular structure of circNUP50 was confirmed using Sanger sequencing.

### RNA fluorescence in situ hybridisation

A fluorescent in situ hybridisation (FISH) reagent (C10910, RiboBio, China) was used to perform RNA-FISH according to the manufacturer’s instructions. RiboBio designed and synthesised the FISH probes for CircNUP50 and hsa-miR-197-3p. All images were captured using a Philips confocal microscope.

### 5-Ethynyl-20-deoxyuridine, cell cycle, and apoptosis assays

Cell proliferation was evaluated using the 5-ethynyl-20-deoxyuridine (EdU) assay kit (No. C0071 s, Beyotime, Beijing, China). Cells (1 × 10^5^) were maintained in 96-well plates. After 48 h, 100 μL of EdU was added to the culture for 2 h. The cells were incubated with 4% formaldehyde, followed by 0.3% Triton X-100 for 10 min. EdU-positive cells were analysed using fluorescence microscopy (Olympus, Tokyo, Japan) with a 20 × objective lens.

We used a cell cycle kit (No. C1052, Beyotime) according to the manufacturer's guidelines for cell cycle analysis on a flow cytometer (Beckman Coulter, Brea, CA, USA) after treatment with RNase A (0.1 mg/mL) and propidium iodide (PI, 0.05 mg/mL; Biotech, Beijing, China) for 30 min at 37 °C.

Apoptosis was assessed using an Annexin V-FITC/PI Apoptosis Detection Kit (No. A211, Vazyme). The samples were analysed using a flow cytometer.

### RNA binding protein immunoprecipitation, RNA pulldown, and dual-luciferase reporter assays

A tagged RNA affinity purification (TRAP) kit (BersinBio, Guangzhou, China) was used for the circRNA pull-down assay. An RNA immunoprecipitation (RIP) kit (BersinBio) was used to perform the RIP experiment as described previously [15].

The dual-luciferase reporter assay involved the use of kits and plasmids supplied by GenePharma. Luciferase activity was measured using the dual-luciferase reporter assay kit (Promega, Madison, WI, USA) on a GloMax Discover Microplate Reader (Promega).

### Psc@DPP preparation and characterisation

Platinum and si-circNUP50 co-delivery nanosystems (Psc@DPP) were established using the emulsification-solvent evaporation technique. Precisely weighed amounts of platinum, methoxy poly(ethylene glycol)-poly(lactide) (mPEG-PLA), and 1,2-dioleoyl-3-trimethylammonium-propane (DOTAP) (separately dissolved in dichloromethane) were combined at a mass ratio of 1:10:5. Sonication was used to incorporate the organic solution into the aqueous phase containing the surfactant sodium cholate. The oil-in-water emulsion was agitated for 24 h after emulsification to evaporate the organic solvent. The nanoparticles were separated by centrifugation at 15000 rpm for 30 min and rinsed with deionised water to eliminate any remaining surfactant. The prepared nanoparticles were incubated with RNA at a mass ratio of 30:1 to obtain Psc@DPP nanoparticles. The particle size and zeta potential of Psc@DPP were characterised using a Zetasizer Nano ZS instrument (Malvern, Worcestershire, UK). Transmission electron microscopy (Talos L120C G2, Thermo Fisher Scientific) and three-dimensional structured illumination microscopy (Delta Vision OMX, Nikon, Japan) were used to characterise nanoparticle morphology.

### Animal studies

Four-week-old female nude mice (BALB/c-nu, HFK Bioscience, Beijing, China) were subcutaneously injected with 0.2 ml of cell suspension containing 1 × 10^6^ stable clone cells (sh-circNUP50-SKOV3/DDP and sh-NC-SKOV3/DDP cells). The mice were divided into four groups, I. Mice were subcutaneously injected with sh-NC-SKOV3/DDP cells (PBS + sh-NC group), and PBS was used as a negative control; II. Mice were subcutaneously injected with sh-circNUP50-SKOV3/DDP cells (PBS + sh-circNUP50 group), and PBS was used as a negative control; III. Mice were subcutaneously injected with sh-NC-SKOV3/DDP cells and treated with platinum (Platinum + sh-NC group); IV. Mice were subcutaneously injected with sh-circNUP50-SKOV3/DDP cells and treated with platinum (Platinum + sh-circNUP50 group). The size of the tumour was assessed weekly. After 4 weeks, the mice were euthanised with intraperitoneal administration of 100 mg/kg pentobarbital sodium (Sigma-Aldrich, St. Louis, MO, USA). The xenograft tumours were surgically removed, and their weights were recorded.

### Constructing nanosystems for drug co-delivery

The Psc@DPP system was used to overcome OC platinum resistance in an OC tumour model. Six-week-old female nude mice were inoculated with 3 × 10^6^ Luc-labelled SKOV3/DDP cells. The mice were anaesthetised, ovarian tissue was removed, cells were injected under the ovarian capsule, and the ovaries were returned to the abdominal cavity and sutured. After 2 weeks, green fluorescence measured by the in vivo imaging system was observed, and the group experiment was conducted. We divided the experiment into four groups, I. Mice were subcutaneously injected with SKOV3/DDP cells (PBS group), and PBS was used as a negative control; II. Mice were subcutaneously injected with SKOV3/DDP cells and treated with platinum (platinum group); III. Mice were subcutaneously injected with SKOV3/DDP cells and treated with platinum and si-circNUP50 (platinum + si-circNUP50 group); IV. Mice were subcutaneously injected with SKOV3/DDP cells and treated with Psc@DPP (Psc@DPP group). Tumour growth was measured after 4 weeks using luminescence signals and in vivo imaging equipment. Mice were euthanised with intraperitoneal administration of pentobarbital sodium (Sigma-Aldrich) at a dosage of 100 mg/kg. After euthanasia, the xenograft tumours were surgically removed.

The safety, biocompatibility, and targeting properties of the Psc@DPP nanosystem were evaluated, mainly by investigating whether Psc@DPP functionally damaged the major organs of the body or induced acute systemic immune-inflammatory response. First, from a safety point of view, the experimental treatment group was mainly divided into three groups: (i) saline-treated group; (ii) DPP-treated group; and (iii) Psc@DPP-treated group. Twenty-four hours after completion of drug administration, blood was obtained from orbital veins of mice and tested. Another key objective was to examine whether Psc@DPP caused physiological damage to the heart, liver, spleen, lungs, kidneys and other organs. For the histomorphological evaluation of the main organs, the treatment groups in the experiments were divided into three groups: (1) saline-treated group; (2) DPP-treated group; and (3) Psc@DPP-treated group. Twenty-four hours after the completion of drug administration, the main organs were stained with haematoxylin–eosin (HE) staining and observed under a microscope. After successful establishment of an orthotopic animal model of ovarian cancer, mice were injected intravenously with DDP, and Psc@DPP (DDP 5 mg/kg) at 1, 4, 8, 12, and 24 h. The mice were euthanised and the tumours were collected and analysed for DDP content using fluorescence quantification.

### Statistical analysis

Statistical analyses were conducted using GraphPad Prism v. 8.01 (La Jolla, CA, USA). The Student's t-test was used to assess and compare the data obtained from the test and control groups. *P*-values < 0.05 indicated statistical significance. The Kaplan‒Meier Plotter online website (http://kmplot.com/analysis/index.php?p=service&cancer=ovar) was used to map the correlation of progression-free survival (PFS), overall survival (OS), and PPS with OC-associated genes. ROC Plotter (https://www.rocplot.org/) was used to validate genes as predictive biomarkers for OC patients receiving platinum-based therapy.

## Results

### CircNUP50 was upregulated in DDP-resistant OC tissues/cells

**Fig. 1:**
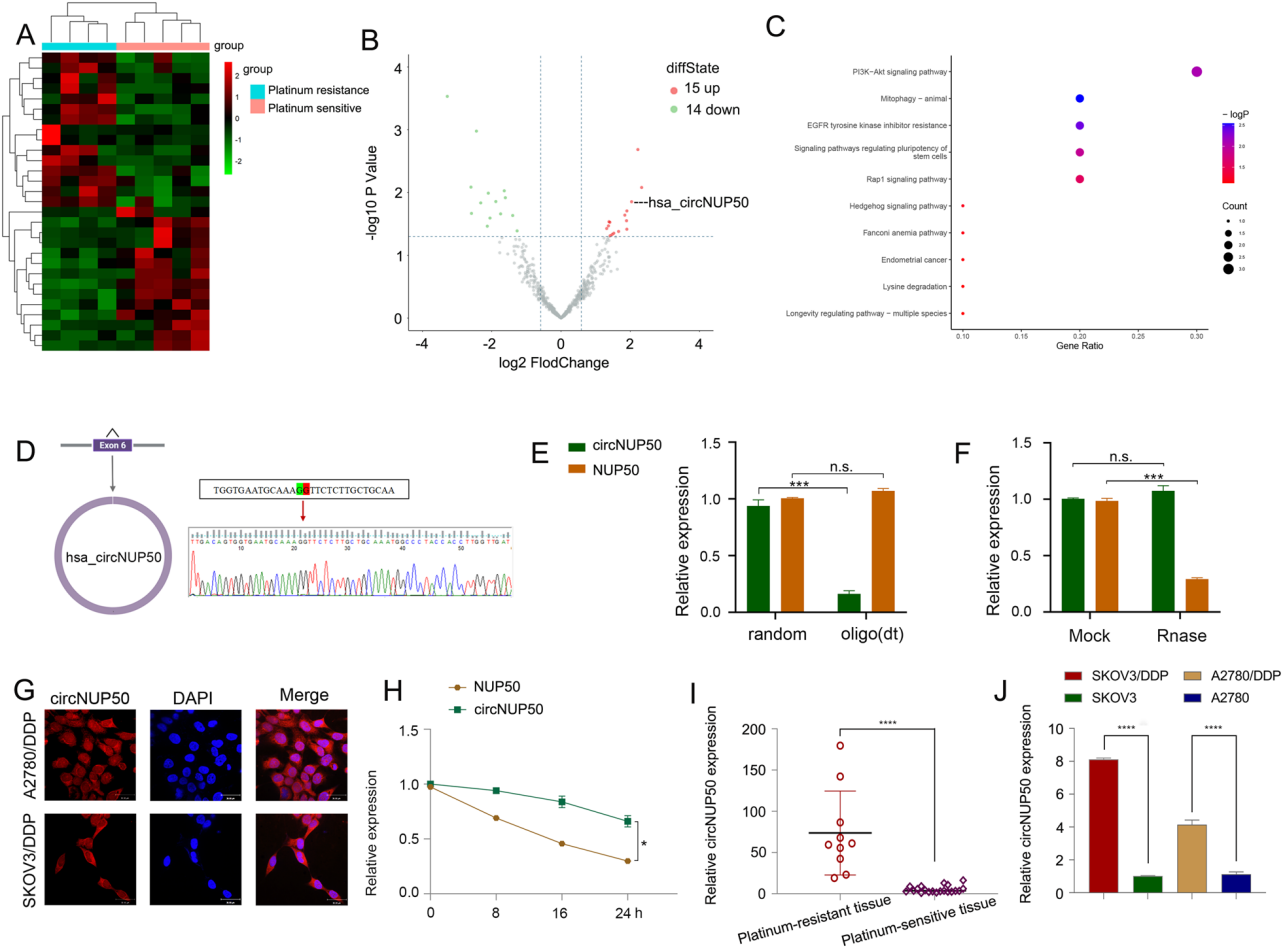
CircNUP50 was upregulated in ovarian cancer (OC) platinum-resistant tissues/cells. **A** Heatmap of circular RNA (circRNA) sequencing of platinum-resistant and platinum-sensitive OC tissues. **B** Scatterplot showed hundreds of circRNA expression differences in platinum-resistant and -sensitive OC tissue. **C** Kyoto Encyclopedia of Genes and Genomes (KEGG) analysis based on circRNA-seq data. **D** The circular structure of circNUP50 was confirmed by amplification with divergent primers, followed by Sanger sequencing, which showed that circNUP50 was generated from exon 6 of the NUP50 gene through back splicing. **E** The circular structure of circNUP50 and the absence of a poly(A) tail of circNUP50 were verified using oligo (dT) and random primers. **F** and **H** RNase R and actinomycin D expression suggested that circNUP50 was more stable than linear NUP50. **G** Fluorescence in situ hybridisation experiments demonstrated that circNUP50 was present in the nucleus and cytoplasm of cells. **I** and **J** The qRT-PCR results revealed circNUP50 overexpression in OC platinum-resistant tissues and cells

Twenty-nine circRNAs differed between platinum-resistant OC and PS tissues (Fig. 1A and B). Kyoto Encyclopedia of Genes and Genomes (KEGG) (Fig. 1C) and Gene Ontology (GO) analysis (Additional file 4: Figure S1A) showed that the differentially expressed circRNAs were associated with the PI3K-Akt signalling pathway, positive growth regulation, and positive autophagy regulation (Additional file 2: Table S4). PI3K/AKT pathway activation promotes OC cell growth, migration, and invasion [16–18]. Inducing autophagy and inactivating the PI3K/AKT/mTOR pathway inhibits the malignant behaviour of OC [19]. In addition, autophagy-related signalling pathways, particularly the PI3K/AKT/mTOR pathways, are upregulated in advanced cancer stages, and the malignant phenotype of the disease reduces autophagy, leading to tumour progression [20]. Thus, the PI3K-Akt pathway and autophagy are associated with tumour progression in OC. Autophagy is one of the causes underlying resistance to many anti-tumour drugs (including DDP). LDLR knockdown could reduce OC tumour growth by inhibiting autophagy associated with the PI3K/AKT/mTOR pathway, and LDLR promoted autophagy-mediated resistance to DDP in OCs associated with the PI3K/AKT/mTOR pathway [21]. Therefore, the PI3K-Akt pathway and autophagy are also associated with platinum resistance in OC. These results suggest that these differences in circRNAs are closely related to tumour progression and drug resistance. We found three upregulated circRNAs with a log2 fold change > 2 (P > 0.05). We then conducted qRT-PCR on the three circRNAs in cells and tissues, and circNUP50 (hsa_circ_0002077, 663 bp) had significant expression differences in cells and tissues. qRT-PCR primers for circNUP50 were prepared (Additional file 4: Figure S1B, and S1C). Sanger sequencing showed that back splicing created circNUP50 from exon 6 of the NUP50 gene (Fig. 1D). qRT-PCR was used to determine whether circNUP50 contains poly(A) tails using oligo (dT) and random primers. If the target gene was not expressed in the oligo (dT) reverse-transcribed cDNA, the RNA is circular and lacks a poly(A) tail. Furthermore, qRT-PCR was used to confirm the circular nature of circNUP50 using oligo (dT) and random primers. circNUP50 expression was significantly reduced compared to NUP50 expression when using random primers, indicating that circNUP50 lacked a poly(A) tail (Fig. 1E). RNase R can only degrade linear RNA, not circRNA, and the RNase R treatment revealed that circNUP50 was resistant to RNase R (Fig. 1F). FISH assays were also performed to determine the location of circRNA. A fluorescent signal corresponding to circNUP50 was expressed in the nucleus and cytoplasm (Fig. 1G). Based on actinomycin D expression, circNUP50 was more stable than linear NUP50 (Fig. 1H). Platinum-resistant OC tissues (Fig. 1I) and cells (Fig. 1J) overexpressed circNUP50.

### CircNUP50 dysregulation contributes to abnormal expression of DDP resistance-associated genes in OC

**Fig. 2:**
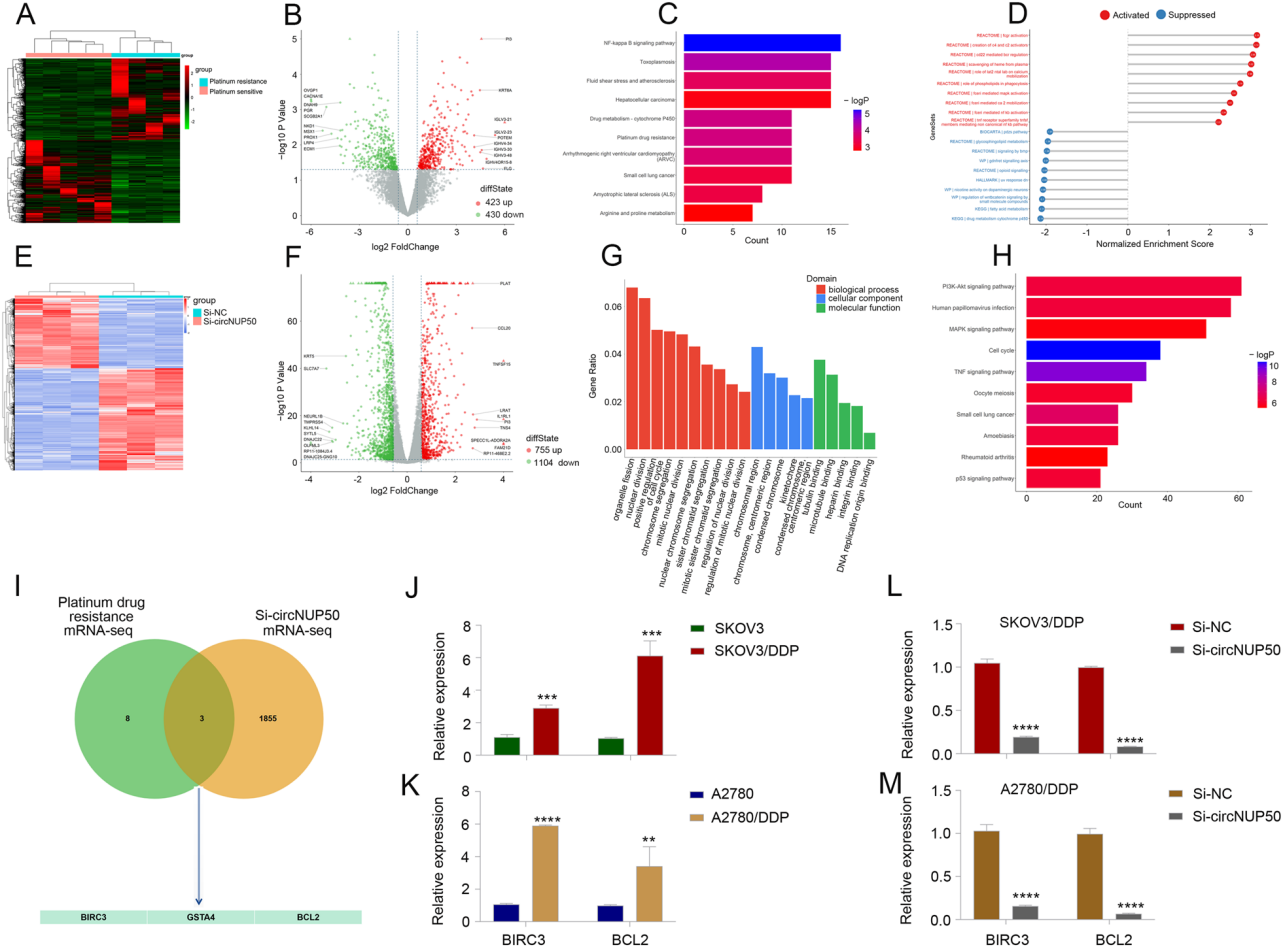
CircNUP50 dysregulation contributes to abnormal expression of platinum resistance-associated genes in OC. **A** and **B** Heatmap and scatterplot of mRNA sequencing of platinum-resistant and -sensitive OC tissues. **C** KEGG analysis based on mRNA-seq data. **D** Gene set enrichment analysis (GSEA) based on mRNA-seq data. **E** and **F** Heatmap and scatterplot of mRNA-seq data of si-circNUP50 SKOV3/DDP and si-NC SKOV3/DDP cells. **G** and **H** KEGG and GSEA based on si-circNUP50 SKOV3/DDP cells and si-NC SKOV3/DDP cell mRNA-seq data. **I** Intersection of mRNA-seq data from OC tissues and si-circNUP50-SKOV3/DDP cells **J** and **K** BIRC3 and BCL2 expression in SKOV3/DDP and A2780/DDP cells. **L** and **M** BIRC3 and BCL2 expression in SKOV3/DDP and A2780/DDP cells transfected with si-circNUP50

We performed mRNA-seq on four platinum-resistant and five PS OC tissues (Fig. 2A and B) and subjected the results to KEGG (Fig. 2C) and gene set enrichment analyses (Fig. 2D) to investigate the aberrant expression of platinum-resistant and circNUP50-related genes in OC The mRNA-seq data were closely associated with cytochrome P450 drug metabolism, platinum drug resistance, and MAPK activation regulation.

We also performed mRNA-seq on si-circNUP50 SKOV3/DDP cells (SKOV3/DDP cells transfected with si-circNUP50) and si-NC SKOV3/DDP cells (Fig. 2E and F). GO (Fig. 2G and KEGG (Fig. 2D) analyses revealed that circNUP50 expression was associated with positive cell cycle regulation in biological processes and the MAPK and p53 signalling pathways. Activating the MAPK pathway reduces the efficacy of platinum drugs in OC [22]. We evaluated mRNA-seq data from OC tissues and si-circNUP50-SKOV3/DDP cells and identified three platinum drug resistance mRNAs, BIRC3, GSTA4, and BCL2, whose function is mediated through circNUP50 (Fig. 2I). Kaplan–Meier analysis revealed that BIRC3 expression was significantly related to OS and PPS (Additional file 5: Figure S2A, S2D, and S2G), GSTA4 expression was significantly related to PFS and OS (Additional file 5: Figure S2B, S2E, and S2H), and BCL2 expression was significantly related to PFS (Additional file 5: Figure S2C, S2F, and S2I). BIRC3 and BCL2 expression was higher in patients resistant to platinum-based treatment and was significantly different from that in patients who did respond to platinum-containing treatment (Additional file 5: Fig. S2J–SL). We then explored BIRC3 and BCL2 expression in DDP-resistant OC cells (SKOV3 and SKOV3/DDP cells, A2780 and A2780/DDP cells), and BIRC3 and BCL2 expression was higher in SKOV3/DDP (Fig. 2J) and A2780/DDP cells (Fig. 2K). Furthermore, BIRC3 and BCL2 expression was reduced in SKOV3/DDP and A2780/DDP cells transfected with si-circNUP50 (Fig. 2L and M). These results suggest that circNUP50 may regulate platinum resistance in OC through BIRC3 and BCL2.

### CircNUP50 regulates the proliferation ability, cell cycle progression, and apoptosis of platinum-resistant OC cells

**Fig. 3:**
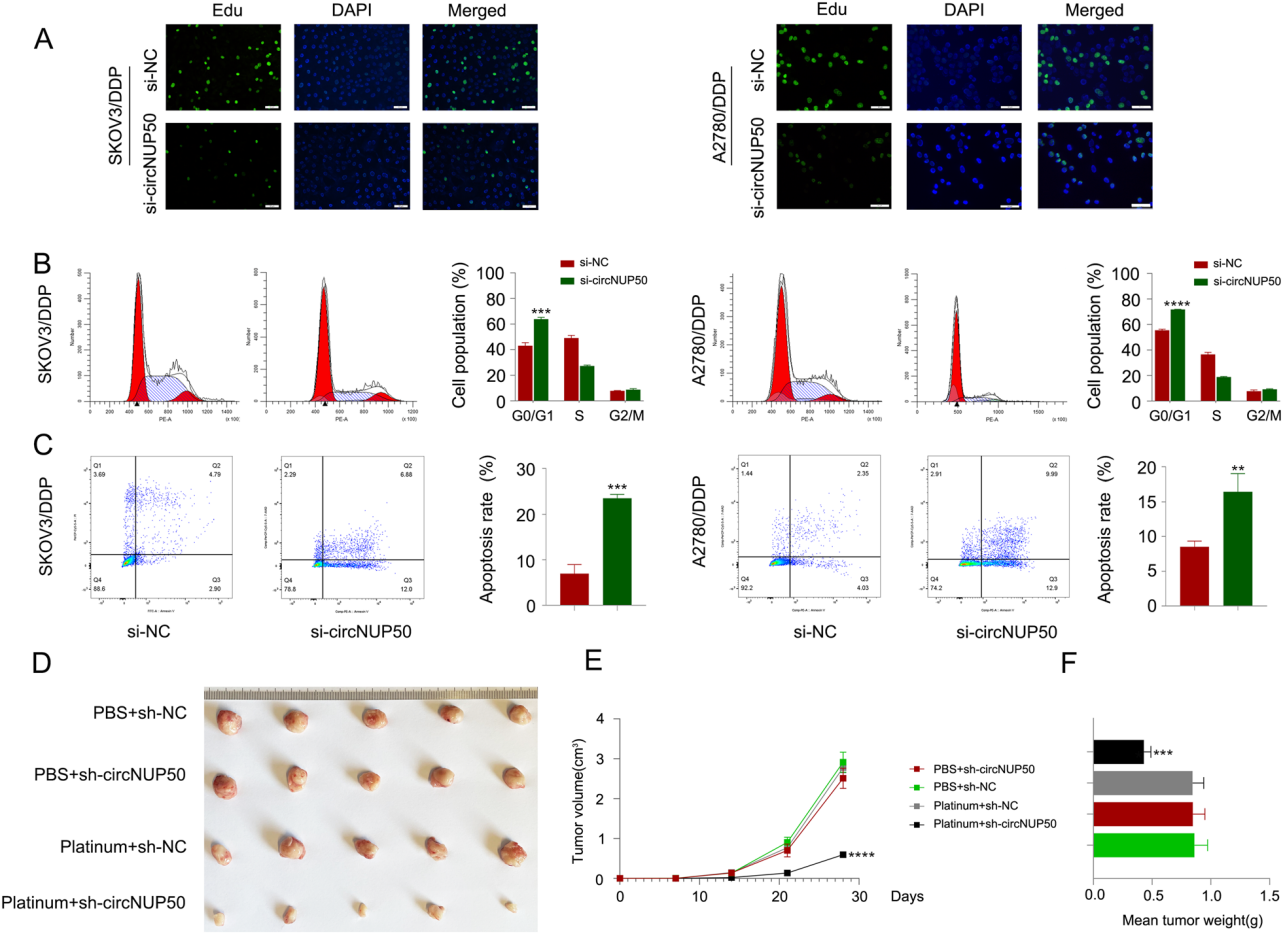
CircNUP50 can regulate the proliferation ability, cell cycle progression, and apoptosis of OC platinum-resistant cells. **A** EdU experiments showed that circNUP50 knockdown reduced the proliferative activity of SKOV3/DDP and A2780/DDP cells. **B** circNUP50 knockdown led to cell cycle arrest at the G0/G1 phase in SKOV3/DDP and A2780/DDP cells. **C** circNUP50 knockdown increased the apoptosis rate in SKOV3/DDP and A2780/DDP cells. **D**–**F** Xenografts formed by SKOV3/DDP cells bearing platinum + sh-circNUP50 exhibited a significantly slower growth rate (**D** and **E**) and lower tumour weight than xenografts from control groups

Based on the mRNA-seq results obtained for si-circNUP50 SKOV3/DDP cells, circNUP50 expression was closely associated with positive regulation of epithelial cell proliferation, positive cell cycle regulation, apoptosis, and drug response (Additional file 3: Table S5). Furthermore, we used an EdU assay to examine cell proliferation and flow cytometry to detect the cell cycle and apoptosis to elucidate the role of circNUP50 in regulating platinum resistance in OC cells. We characterised the phenotypes in SKOV3/DDP and A2780/DDP cells with circNUP50 silencing (si-circNUP50). As si-circNUP50 significantly inhibited circNUP50 expression in platinum-resistant OC cells (SKOV3/DDP and A2780/DDP cells) (Additional file 4: Figure S1D), we chose si-circNUP50 for the cell function experiments. The drug resistance of si-NC SKOV3/DDP, si-NC A2780/DDP, si-circNUP50 SKOV3/DDP, and si-circNUP50 A2780/DDP cells was maintained at a DDP concentration of 6 μM. CircNUP50 expression knockdown could reduce the proliferative activity of SKOV3/DDP and A2780/DDP cells compared to that of control cells (Fig. 3A). Cell cycle experiments showed that circNUP50 expression knockdown significantly induced cell cycle arrest at the G0/G1 phase in SKOV3/DDP and A2780/DDP (Fig. 3B). Apoptosis experiments showed that the circNUP50 knockdown increased the apoptosis rate in SKOV3/DDP and A2780/DDP cells (Fig. 3C).

Subsequently, we validated the role of circNUP50 in mediating resistance to platinum treatment in OC in vivo. We designed four sets of controlled experiments (PBS + sh-NC, PBS + sh-circNUP50, platinum + sh-NC, and platinum + sh-circNUP50), and tumour xenograft data showed no significant difference in tumour size between the two groups of mice treated with PBS; however, downregulating circNUP50 in DDP-resistant cells (platinum + sh-NC group vs. platinum + sh-circNUP50 group) significantly reduced xenograft tumour growth and sensitised the cells to DDP treatment (Fig. 3D). Moreover, xenograft tumours formed by SKOV3/DDP cells carrying sh-circNUP50 displayed substantially slower growth rates (Fig. 3E) and lower tumour weights (Fig. 3F). These findings suggest that circNUP50 contributes to platinum resistance in OC by promoting cell proliferation, affecting the cell cycle, and reducing apoptosis.

### CircNUP50 mediates platinum resistance in OC by regulating p53 ubiquitination by binding to UBE2T

**Fig. 4:**
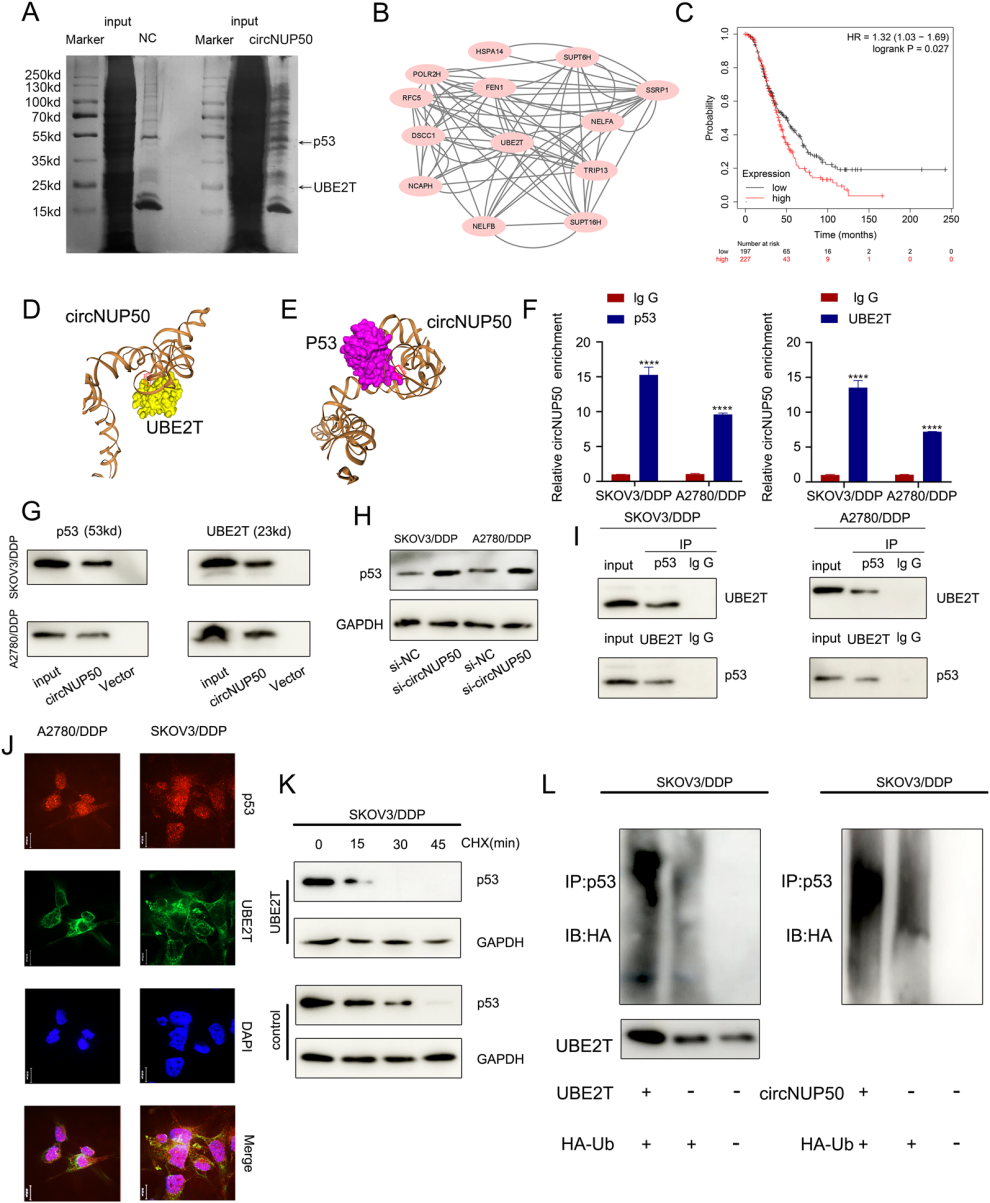
CircNUP50 mediates platinum resistance in OC by regulating p53 ubiquitination by binding to UBE2T. **A** Silver staining of the RNA pull-down showed that circNUP50 bound to UBE2T and p53. **B** Protein–Protein Interaction (PPI) network analysis based on mass spectrometry data of circNUP50-binding proteins. **C** UBE2T expression with respect to OS of OC patients treated with cisplatin-containing regimens. **D** and **E** Molecular simulation results supported that circNUP50 could accurately dock with UBE2T or p53. **F** and **G** RNA binding protein immunoprecipitation (RIP) analysis and circRNA pull-down showed that p53 and UBE2T could interact with circNUP50. **H** Enhanced p53 expression levels after circNUP50 knockdown. **I** Co-immunoprecipitation experiments showed that p53 and UBE2T could bind to each other. **J** Co-localisation experiments showed that p53 and UBE2T were co-localised in the cytoplasm of SKOV3/DDP and A2780/DDP cells. **K** Cycloheximide (CHX) chase assays showed that the half-life of p53 in SKOV3/DDP cells was reduced when UBE2T was overexpressed. **L** The ubiquitination experiment showed that UBE2T overexpression and circNUP50 in SKOV3/DDP enhanced p53 ubiquitination

To explore the proteins bound to circNUP50, we performed silver staining of the RNA pull-down (Fig. 4A), followed by comprehensive identification of RNA-binding proteins using mass spectrometry (ChIRP-MS) (Additional file 6: Figure S3A and S3B). CircNUP50 can bind hundreds of proteins relative to controls, and PPI (Supplementary Fig. 3C) and GO analyses of these bound proteins (Additional file 6: Figure S3D) were performed. These analyses showed that the binding proteins could mediate ubiquitination modifications in the context of biological processes, cellular components, and molecular functions. GO analysis of the circRNA-seq, mRNA-seq on si-circNUP50 SKOV3/DDP cells, and mass spectrometry showed that circNUP50 regulates ubiquitination and plays an essential role in platinum resistance of OC. Therefore, Ub‐conjugating enzyme E2T (UBE2T), a crucial ubiquitin-conjugating enzyme among the binding proteins, attracted our attention (Fig. 4B). High UBE2T expression was significantly associated with poor OS in OC patients treated with DDP-containing regimens (Fig. 4C). UBE2T promotes hepatocellular carcinoma cell growth through p53 ubiquitination [23]. Interestingly, GO and KEGG analysis of the si-circNUP50 SKOV3/DDP cells mRNA-seq data showed that circNUP50 expression regulates p53-related pathways. Therefore, we hypothesised that circNUP50 accelerates UBE2T ubiquitination regulation of p53 through molecular scaffolding.

We investigated whether circNUP50 interacts with UBE2T or p53, and its secondary structure was assembled in Mfold (v. 2.3). circNUP50 can dock perfectly with UBE2T and p53 (Fig. 4D and E). We performed RIP analysis using anti-UBE2T and anti-p53 antibodies to further characterise the interaction of circNUP50 with UBE2T and p53. p53 and UBE2T interacted with circNUP50 in SKOV3/DDP and A2780/DDP cells (Fig. 4F). TRAP-western blotting showed that p53 and UBE2T could bind to circNUP50-MS2 (Fig. 4G). Furthermore, western blotting verified that circNUP50 accelerates p53 ubiquitination through the molecular scaffold and p53 expression in SKOV3/DDP and A2780/DDP cells increased after silencing circNUP50 (Fig. 4H).

Next, we verified the ubiquitination regulation of p53 by UBE2T in SKOV3/DDP cells. Co-immunoprecipitation (Co-IP) showed that p53 and UBE2T could bind to each other in SKOV3/DDP and A2780/DDP cells (Fig. 4I). Co-localisation experiments showed that p53 and UBE2T proteins co-localised in the cytoplasm of SKOV3/DDP and A2780/DDP cells (Fig. 4J). Cycloheximide (CHX) chase assays showed that the half-life of p53 in SKOV3/DDP cells was reduced when UBE2T was overexpressed (compared to that in the control) (Fig. 4K). The ubiquitination experiment showed that UBE2T overexpression in SKOV3/DDP enhanced p53 ubiquitination. circNUP50 overexpression also enhanced p53 ubiquitination (Fig. 4L). Therefore, circNUP50 can bind p53 and UBE2T simultaneously and mediate platinum resistance in OC by binding to UBE2T to regulate p53 ubiquitination.

### CircNUP50 upregulates G3BP1 expression by acting as a miR-197-3p sponge

**Fig. 5:**
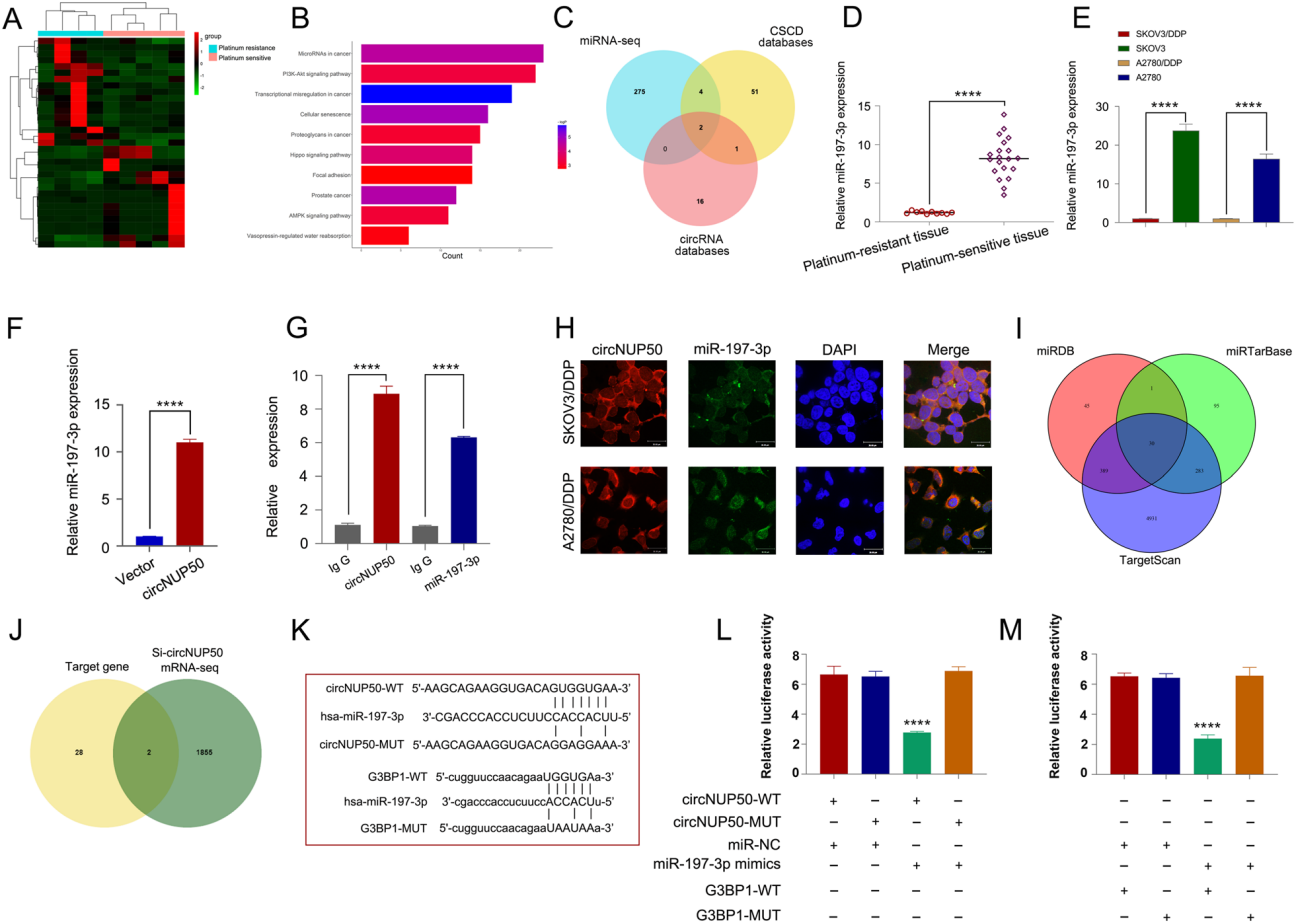
CircNUP50 upregulates G3BP1 by acting as a miR-197-3p sponge. **A** Heatmap of miRNA sequencing of platinum-resistant and PS OC tissues. **B** KEGG analysis based on miRNA-seq data. **C** Identification of miRNAs that bind to circNUP50. **D** miR-197-3p expression is significantly different in platinum-resistant and PS OC tissues. **E** miR-197-3p expression is significantly different in platinum-resistant and PS cells. **F** circRNA pull-down assay showed that miR-197-3p could specifically bind to circNUP50. **G** RIP experiments showed increased circNUP50 and miR-197-3p enrichment in the Ago2 group compared to that in the IgG group. **H** RNA FISH experiments showed that circNUP50 and miR-197-3p were co-localised in the cytoplasm. **I** Identification of specific target genes of miR-197-3p from three databases. **J** After combining si-circNUP50 mRNA-seq data, two target genes of miR-197-3p were identified. **K**–**M** Luciferase activity was significantly inhibited in cells co-transfected with miR-197-3p mimic and wild-type luciferase reporter genes (circNUP50-WT and G3BP1-WT) compared to that in the controls

circRNA is a molecular sponge that adsorbs miRNAs to regulate the expression of downstream genes. We performed miRNA-seq analyses of platinum-resistant and PS OC tissues to investigate whether miRNAs can be sponged by circNUP50 (Fig. 5A). KEGG analysis of the data showed that miRNAs were enriched in the platinum resistance-associated PI3K-Akt and AMPK signalling pathways (Fig. 5B). The CSCD (http://gb.whu.edu.cn/CSCD/) and circRNA databases (https://circinteractome.nia.nih.gov/) were used to predict miRNA binding to circNUP50 and combined with the miRNA-seq data from OC tissues; these findings indicate that only miR-197-3p and miR-1296-5p could bind to circNUP50 (Fig. 5C). qRT-PCR showed that miR-197-3p expression was higher in OC PS tissues and significantly different than in OC platinum-resistant tissues (Fig. 5D). Similarly, miR-197-3p expression significantly differed in platinum-resistant and PS cells (Fig. 5E). By contrast, miR-1296-5p expression in platinum-resistant and PS cells was low and less significant than that of miR-197-3p (Additional file 6: Figure S3E). Therefore, we selected miR-197-3p for the follow-up study. miR-197-3p can specifically bind circNUP50 based on a circRNA pull-down assay (Fig. 5F). RIP experiments showed increased circNUP50 and miR-197-3p enrichment in the Ago2 group compared to that in the IgG group (Fig. 5G). RNA FISH experiments showed that circNUP50 and miR-197-3p co-localised in the cytoplasm (Fig. 5H).

To elucidate the function of miR-197-3p, we used an EdU assay to examine the proliferation of platinum-resistant cells; we used flow cytometry for cell cycle analysis and apoptosis estimation. The proliferation of SKOV3/DDP (Additional file 7: Figure S4A) and A2780/DDP cells (Additional file 7: Figure S4B) was reduced when miR-197-3p was overexpressed. Cell cycle assays showed that SKOV3/DDP (Additional file 7: Figure S4C) and A2780/DDP cells (Additional file 7: Figure S4D) were arrested in the G0/G1 phase of the cell cycle when miR-197-3p was overexpressed. Apoptosis experiments revealed that the apoptosis rate of SKOV3/DDP (Additional file 7: Figure S4E) and A2780/DDP (Additional file 7: Figure S4F) cells was increased after overexpressing miR-197-3p. Furthermore, the function of anti-miR-197-3p was rescued by the decrease in circNUP50 levels.

In addition, we reviewed the miRDB, miRTarBase, and TargetScan databases to find specific miR-197-3p target genes and 30 target genes were discovered (Fig. 5I). G3BP1 and FRMPD3 were target genes when the si-circNUP50 mRNA-seq data were combined (Fig. 5J). The OS, PFS, and PPS of G3BP1 (Additional file 8: Figure S5A–C) and FRMPD3 (Additional file 8: Figure S5F–SH) in platinum-containing treated OC were mapped on the Kaplan‒Meier Plotter online website. G3BP1 expression was higher in patients resistant to platinum-containing therapy and differed significantly from that of patients who responded to platinum-containing therapy (Additional file 8: Figure S5D and S5E). By contrast, FRMPD3 expression was not significantly different in patients who responded or were resistant to platinum-containing therapy (Additional file 8: Figure S5I and SJ). Therefore, G3BP1 was identified as a downstream target gene. Luciferase reporter assay showed that miR-197-3p could bind to circNUP50 and G3BP1. When miR-197-3p mimic and wild-type luciferase reporter genes (circNUP50-WT and G3BP1-WT) were co-transfected into cells, luciferase activity significantly decreased (compared to that in the controls) (Fig. 5K–M). qRT-PCR revealed a significant decrease in G3BP1 expression upon overexpressing the miR-197-3p mimic (Additional file 8: Figure S5K). In contrast to sh-circNUP50 transfection, anti-miR-197-3p transfection in SKOV3/DDP cells elevated G3BP1 expression (Additional file 8: Figure S5L). These experiments demonstrated that circNUP50 could act as a miR-197-3p sponge to upregulate G3BP1 to mediate platinum resistance in OC.

### G3BP1 mediates p53 ubiquitination to regulate platinum resistance in OC

**Fig. 6:**
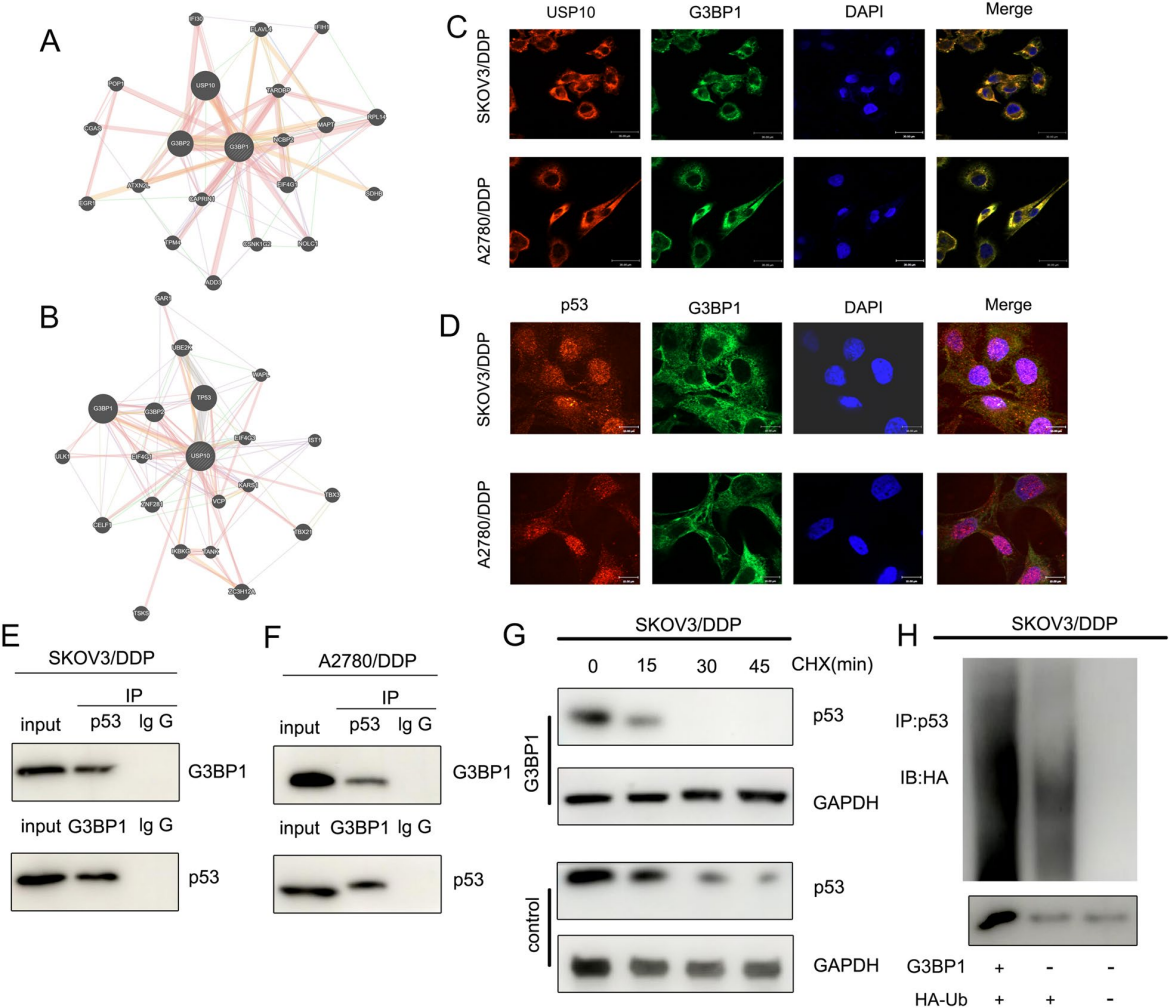
G3BP1 mediates p53 ubiquitination to regulate platinum resistance in OC. **A** PPI analysis revealed that G3BP1 interacts with USP10. **B** PPI analysis results showed that USP10 interacted with G3BP1 and p53. **C** and **D** RNA FISH showed that G3BP1 co-localised with USP10 and p53 in the cytoplasm. **E** and **F** Co-IP demonstrated that p53 protein and G3BP1 bound to each other in SKOV3/DDP and A2780/DDP cells. **G** CHX chase assays showed that the half-life of p53 in SKOV3/DDP cells was reduced when G3BP1 was overexpressed compared to that in the control group. **H** Ubiquitination assay showed that the G3BP1 overexpression enhanced the ubiquitinated p53 expression in SKOV3/DDP cells

We performed PPI analysis and found that G3BP1 interacted with USP10 (Fig. 6A), and G3BP1 was involved in the ubiquitination of p53 by USP10 [24]. Subsequent PPI analysis verified that G3BP1 interacts with USP10 and TP53 (Fig. 6B). RNA FISH showed that G3BP1 co-localised with USP10 and p53 in the cytoplasm (Fig. 6C and D). Co-IP demonstrated that p53 and G3BP1 were bound to each other in SKOV3/DDP and A2780/DDP cells (Fig. 6E and F). CHX chase assays showed that the half-life of p53 in SKOV3/DDP cells was reduced when G3BP1 was overexpressed compared to that in the control group (Fig. 6G). Ubiquitination assays showed that G3BP1 overexpression enhanced p53 ubiquitination in SKOV3/DDP cells (Fig. 6H). These experiments suggest that G3BP1 can mediate p53 ubiquitination to regulate platinum resistance in OC.

### Synergistic codelivery of si-circNUP50 and platinum using nanosystems to overcome platinum resistance in an OC tumour model

**Fig. 7:**
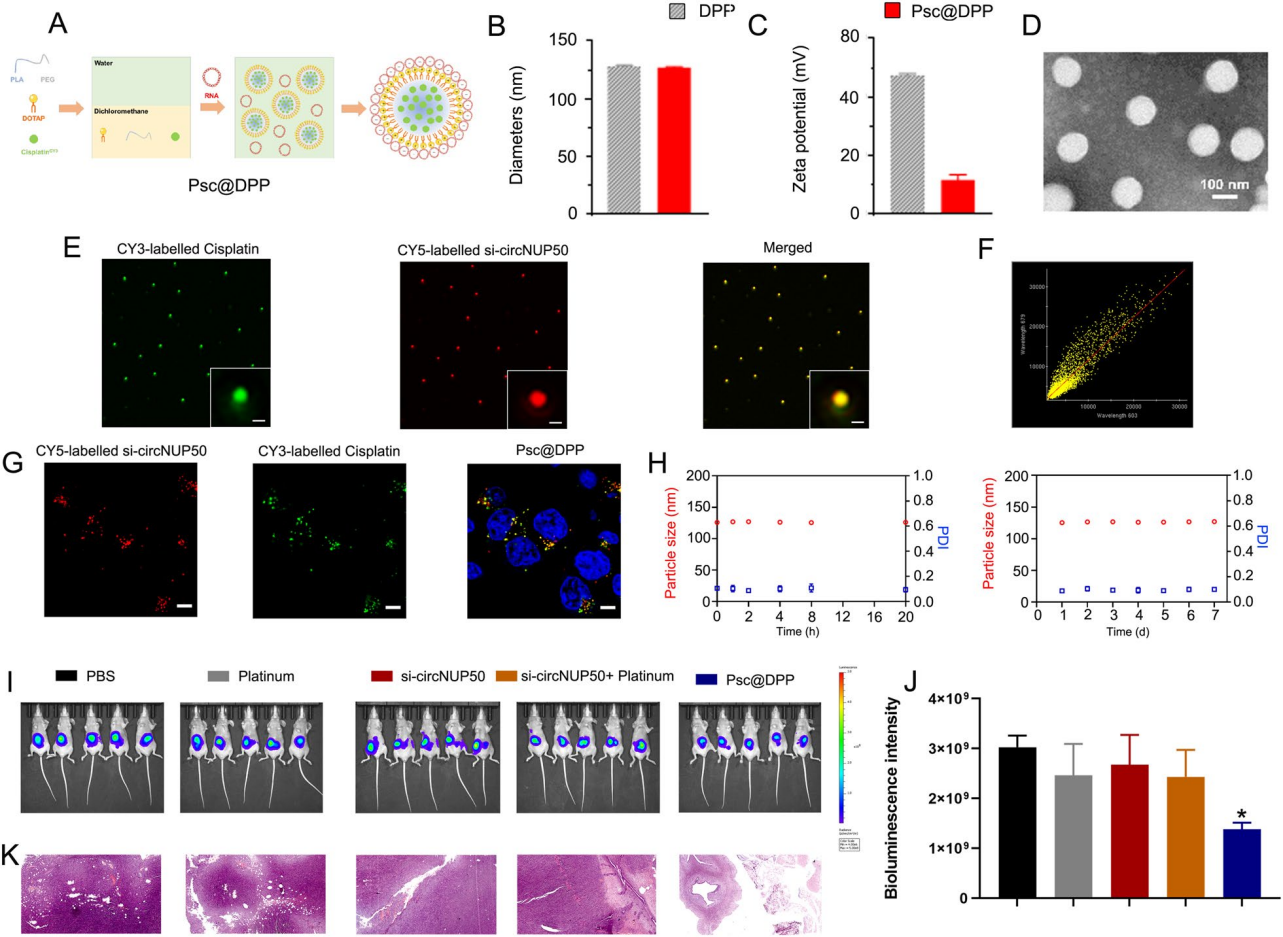
si-circNUP50 and platinum co-delivery using nanosystems to overcome platinum resistance in an OC tumour model. **A** Structure of the Psc@DPP co-delivery nanosystem. **B** and **C** The particle size of Psc@DPP is 150 nm, and the zeta potential of Psc@DPP is + 15 mV. **D** The transmission electron microscopy image of Psc@DPP. **E** Fluorescence signals corresponding to platinumCy3 and si-circNUP50Cy5 on Psc@DPP were statistically analysed using Pearson’s correlation test. **F** The Pearson’s correlation coefficient of platinumCy3 and si-circNUP50Cy5 on Psc@DPP was 0.86. **G** After co-incubating Psc@DPP with ovarian tumour cells for 4 h, Psc@DPPs delivered platinumCy3 and si-circNUP50Cy5 into OC cells. **H** The serum stability of Psc@DPP within 24 h and the storage stability of the nanoparticles within 7 days was good. **I** and **J** The fluorescence intensity was significantly lower in the Psc@DPP group compared than in the PBS and platinum groups, whereas there was no significant change in fluorescence intensity in the platinum + si-circNUP50 group. **K** Haematoxylin and eosin staining of OC tissues revealed decreased tumour area in the Psc@DPP group when compared with that in the other groups

A platinum and si-circNUP50 co-delivery nanosystem (Psc@DPP) were prepared to treat platinum-resistant OC in an orthotopic animal model (Fig. 7A). Psc@DPP comprises polyethylene glycol monomethyl ether-polylactic acid (mPEG-PLA) and DOTAP. As platinum chemotherapeutic drugs are hydrophobic, they can be loaded within the hydrophobic block PLA of Psc@DPP. Due to its positive charge, DOTAP can load si-circNUP50 onto Psc@DPP through electrostatic adsorption. The particle size of Psc@DPP is 150 nm, and the zeta potential is + 15 mV (Fig. 7B and C). Transmission electron microscopy image of Psc@DPP is presented in Fig. 7D. To verify the co-loading of platinum and si-circNUP50 on Psc@DPP, Cy3-labelled platinum and Cy5-labelled si-circNUP50 were loaded onto Psc@DPP. The fluorescence signals of platinumCy3 and si-circNUP50Cy5 on Psc@DPP were photographed with three-dimensional structured illumination microscopy. The fluorescence signals of platinumCy3 and si-circNUP50Cy5 on Psc@DPP were statistically analysed using Pearson’s correlation test (Fig. 7E). The Pearson’s correlation coefficient of platinumCy3 and si-circNUP50Cy5 on Psc@DPP was 0.86 (Fig. 7F). After co-incubating Psc@DPP with ovarian tumour cells for 4 h, Psc@DPPs delivered platinumCy3 and si-circNUP50Cy5 into ovarian tumour cells (Fig. 7G). The serum stability of Psc@DPP within 24 h and the storage stability of the nanoparticles within 7 days was good (Fig. 7H).

**Fig. 8:**
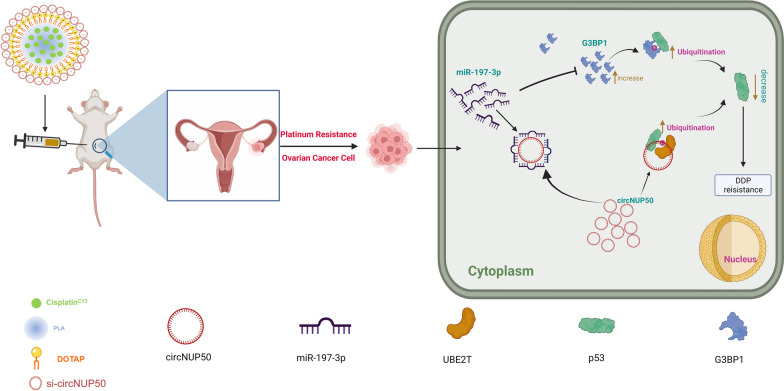
Graphical Abstract. Interference with circNUP50-regulated p53 ubiquitination reverses platinum resistance in ovarian cancer (OC), and synergistic co-delivery of si-circNUP50 and platinum using nanosystems can overcome platinum resistance in an OC tumour model

We constructed an OC platinum-resistant orthotopic animal model to explore the potential anti-tumour effects of Psc@DPP in vivo. We observed that the fluorescence intensity was significantly lower in the Psc@DPP group than in the PBS and platinum groups. By contrast, no significant change in the fluorescence intensity in the platinum + si-circNUP50 group was observed (Fig. 7I and J). HE staining of OC tissues showed decreased tumour area in the Psc@DPP group compared to that in the other groups (Fig. 7K). The safety, biocompatibility, and targeting properties of the Psc@DPP nanosystem were evaluated. Psc@DPP had a good safety profile (Additional file 9: Figure S6A) and caused no damage to major organs, including the heart, liver, spleen, lungs, and kidneys (Additional file 9: Figure S6B), with better targeting properties within tumour tissues (Additional file 9: Figure S6C). In addition, the potential anti-tumour effects of Psc@DPP were explored at the cellular level in vitro. In the Psc@DPP experimental group, the proliferation of SKOV3/DDP and A2780/DDP cells (Additional file 10: Figure S7A) was attenuated, SKOV3/DDP and A2780/DDP cells (Additional file 10: Figure S7B, p < 0.05) were arrested in the G0/G1 phase of the cell cycle, and the apoptosis rates of SKOV3/DDP and A2780/DDP cells were increased (Additional file 10: Figure S7C, p < 0.05). The results suggest that Psc@DPP effectively overcame platinum resistance in an OC tumour model and offers a novel approach for treating platinum-resistant OC using si-circNUP50 (Fig. 8).

## Discussion

Most patients with OC acquire DDP resistance, which is the primary cause of short survival and poor prognosis. Therefore, determining the mechanisms of platinum resistance in patients with OC and discovering novel molecular therapeutic targets to reverse platinum resistance is crucial. We are the first to report that circNUP50 is associated with platinum resistance in OC, and that it regulates p53 ubiquitination by binding to UBE2T. Furthermore, circNUP50 can act as a miR-197-3p sponge to mediate p53 ubiquitination by upregulating G3BP1 to regulate platinum resistance. The constructed Psc@DPP co-delivery nanosystem could treat platinum resistance in OC. These findings reveal a novel molecular mechanism by which circNUP50 regulates platinum resistance in OC.

In eukaryotic cells, intracellular protein ubiquitination is widespread and is an essential posttranslational modification, and most intracellular proteins are degraded via the ubiquitin-dependent proteasome pathway [25]. UBE2T is an E2 enzyme with E2 and E3 ligase activity acting as an oncogene in various tumour types [26]. Furthermore, UBE2T plays a key role in cell cycle progression and tumorigenesis [27]. For example, UBE2T-mediated p53 degradation confers gemcitabine resistance in pancreatic cancer by promoting pyrimidine biosynthesis and alleviating replication stress [28]. In this study, circNUP50 bound to UBE2T and p53, and p53 and UBE2T could also interact with circNUP50. Furthermore, p53 and UBE2T could bind to each other. p53 and UBE2T proteins co-localised in the cytoplasm of SKOV3/DDP and A2780/DDP cells. UBE2T overexpression accelerated p53 degradation. UBE2T and circNUP50 overexpression enhanced p53 ubiquitination in SKOV3/DDP cells. Our findings show that circNUP50 functions as a scaffold to facilitate p53 ubiquitination. These results suggest that circNUP50 can bind to p53 and UBE2T and mediate platinum resistance in OC by binding the UBE2T protein to regulate p53 ubiquitination.

Another important function of circRNA is to act as a miRNA sponge to regulate cancer development. In this study, circNUP50 could adsorb miR-197-3p to act as its sponge and upregulate G3BP1 to mediate p53 ubiquitination, thereby modulating platinum resistance in OC. P53 aggregation has been reported as a potential therapeutic target for reversing chemoresistance, which is critical for improving the response of patients with OC to chemotherapy, thereby improving their survival rates [29]. Resveratrol binds to G3BP1 and blocks the G3BP1/USP10 interaction, enhancing USP10-mediated p53 deubiquitination and increasing p53 expression [24]. In our study, circNUP50 was a miR-197-3p sponge that regulates G3BP1 expression in the cytoplasm and mediates p53 ubiquitination to modulate platinum resistance in OC. Only a few studies have reported that platinum resistance in OC is mediated by modulating the p53 ubiquitination pathway. For example, Abedini et al. [30] reported that Akt generates platinum resistance in OC by regulating DDP-induced, p53-dependent ubiquitination of FLIP. In addition, Fn14 has been reported to overcome DDP resistance in high-grade plasma OC by promoting Mdm2-mediated ubiquitination p53-R248Q degradation [31]. However, no studies have reported that circRNAs are essential molecular targets for regulating the p53 ubiquitination pathway to mediate platinum resistance in OC, and our data provide a previously unrecognised mechanism of p53 degradation and novel evidence for the involvement of circNUP50 in protein metabolism. Furthermore, the present study explored two biological functions of circNUP50 to regulate p53 ubiquitination and comprehensively elucidated its role in platinum resistance in OC.

Another innovative achievement of this study is the design and synthesis of a co-delivery nanosystem (Psc@DPP) that can adsorb platinum and si-circNUP50 to overcome platinum resistance in OC. Several studies show that nanodelivery systems encapsulating drugs have good targeting ability and disease therapeutic efficacy [32–34]. As siRNA has no tumour-targeting ability and siRNA synthesised in vitro is unstable and may be degraded before reaching the tumour site in vivo, siRNA for tumour therapy still has significant drawbacks. Therefore, nanodrug delivery systems have become important for improving drug solubility and tumour-targeting ability [35]. Considering the abovementioned influencing factors and given the therapeutic value of circNUP50 upregulation in OC platinum resistance, synthesising an effective gene therapy vector to address the ability of siRNA and platinum to reach tumour sites and produce synergistic therapeutic effects simultaneously is essential. Therefore, we constructed a nanodrug delivery platform in this study. We observed a significant reduction in ovarian tumour areas in the Psc@DPP treatment group (compared to those in the platinum + si-circNUP50 group). These results suggested that Psc@DPP effectively overcame platinum resistance in an OC tumour model and provides a novel idea for treating platinum-resistant OC by si-circNUP50.

However, this study had some limitations. We used a large sample of public data for survival analysis to compensate for the paucity of real-world tissue specimen validation to verify the survival prediction indicted by the expression levels of UBE2T and G3BP1 genes in platinum-resistant OC patients.

## Conclusion

Our study reveals a novel mechanism by which circNUP50 mediates platinum resistance in OC by modulating p53 ubiquitination, suggesting that antagonising circNUP50 expression could be a therapeutic strategy to overcome DDP resistance in OC.

### Supplementary Information

**Additional file 1: Table S1**. Clinical information of OC samples. **Table S2**. The information of sequence. **Table S3. **The sequence of primers.

**Additional file 2. Table S4**. KEGG and GO analysis based on circRNA-seq data.

**Additional file 3. Table S5**. KEGG and GO analysis based on the mRNA-seq results from si-circNUP50 SKOV3/DDP cells and si-NC SKOV3/DDP cells.

**Additional file 4: Figure S1**: (A) Gene Ontology (GO) analysis based on circular RNA-sequencing (circRNA-seq) data. (B) and (C) Melt and separation curves display the primers used for circNUP50 amplification in qRT-PCR experiments. (D) qRT-PCR showing that si-circNUP50 could significantly inhibit circNUP50 expression in OC cells.

**Additional file 5: Figure S2**: (A), (D), (G) Association of BIRC3 expression with progression-free survival (PFS), overall survival (OS), and PPS in patients with OC. (B), (E), (H) Association of GSTA4 expression with PFS, OS and PPS in patients with OC. (C), (F), (I) Association of BCL2 expression with PFS, OS and PPS in patients with OC. (J), (K), (L) Correlation of the three gene expressions (BIRC3, GSTA4, and BCL2) with the efficacy of platinum-containing therapy.

**Additional file 6: Figure S3**: (A) and (B) Mass spectrometry results of circNUP50-binding proteins. (C) PPI analysis based on mass spectrometry data of circNUP50-binding proteins. (D) GO analysis based on mass spectrometry data. (E) The expression of miR-1296-5p in platinum-resistant and platinum-sensitive (PS) cells was not as significant as that of miR-197-3p.

**Additional file 7: Figure S4**: (A) and (B) 5-ethynyl-20-deoxyuridine (EdU) experiments showed that the proliferation of SKOV3/DDP and A2780/DDP cells reduced when miR-197-3p was overexpressed. (C) and (D) Cell cycle analysis showed that SKOV3/DDP and A2780/DDP cells were arrested in the G0/G1 phase of the cell cycle when miR-197-3p was overexpressed. (E) and (F) Apoptosis experiments showed that the apoptosis rate of SKOV3/DDP and A2780/DDP cells was increased after miR-197-3p overexpression.

**Additional file 8: Figure S5**: (A–C) Association of G3BP1 expression with OS, PFS and PPS in patients OC. (D) and (E) G3BP1 expression significantly differed from patients who responded to platinum-containing therapy. (F–H) Association of FRMPD3 expression with OS, PFS and PPS in patients with OC. (I) and (J) FRMPD3 expression was not significantly different in patients who responded to platinum-containing therapy. (K) qRT-PCR showed that G3BP1 expression was significantly decreased when miR-197-3p mimics were overexpressed. (L) In contrast to sh-circNUP50 transfection, anti-miR-197-3p transfection in SKOV3/DDP cells resulted in elevated G3BP1 expression.

**Additional file 9: Figure S6**: Safety (A), organ damage (B) and tumour targeting (C) properties of the Psc@DPP nanosystem.

**Additional file 10: Figure S7**: Potential anti-tumour effects of the Psc@DPP nanosystem on cell proliferation (A), cell cycle (B), and cell apoptosis (C).

## Data Availability

All the data of the current study are available from the corresponding authors upon reasonable request.

## Abbreviations

OC: Ovarian Cancer
circRNA: Circular RNA
FISH: Fluorescence In Situ Hybridization
qRT–PCR: Quantitative Reverse-Transcription Polymerase Chain Reaction
RIP: RNA Immunoprecipitation
ChIRP-MS: Comprehensive Identification of RNA-binding Proteins by Mass Spectrometry
miRNA: MicroRNA
TRAP: Tagged RNA Affinity Purification
GO: Gene Ontology
KEGG: Kyoto Encyclopedia of Genes and Genomes
GSEA: Gene Set Enrichment Analysis
OS: Overall Survival
PPS: Progression Free Survival
EdU: 5-Ethynyl-20-deoxyuridine
DPP: DOTAP‑mPEG‑PLA
