# Supplementary material for: CircNUP50 is a novel therapeutic target that promotes cisplatin resistance in ovarian cancer by modulating p53 ubiquitination
Source: J Nanobiotechnology. 2024 Jan 19;22:35. doi: 10.1186/s12951-024-02295-w (PMC10799427; doi:10.1186/s12951-024-02295-w)
Supplement: Supplementary file 1 — Additional file 1: Table S1. Clinical information of OC samples. Table S2. The information of sequence. Table S3. The sequence of primers. [file 12951_2024_2295_MOESM1_ESM.docx]

**Additional file1: Table S1. Clinical information of OC samples**

| Experiments | Clinical characteristics | | N (%) |
| --- | --- | --- | --- |
| Clinical information of OC samples in qPCR experiment | Age | <60 | 21 (70) |
|  |  | ≥60 | 9 (30) |
|  | Platinum treatment status | sensitive | 20 (66.7) |
|  |  | resistance | 10 (33.3) |
|  | Pathological type | high grade serous ovarian cancer | 30 (100) |
|  | Stage | I-II | 4 (13.3) |
|  |  | III-IV | 26 (86.7) |
| Clinical information of OC samples in sequencing experiments | Age | <60 | 5 (55.6) |
|  |  | ≥60 | 4 (44.4) |
|  | Platinum treatment status | sensitive | 5 (55.6) |
|  |  | resistance | 4 (44.4) |
|  | Pathological type | high grade serous ovarian cancer | 9 (100) |
|  | Stage | I-II | 1 (11.1) |
|  |  | III-IV | 8 (88.9) |

**Additional file 1: Table S2. The information of sequence**

| si-circNUP50-1 | GCAAAGGUUCUCUUGCUGCTT  GCAGCAAGAGAACCUUUGCTT |
| --- | --- |
| sh-circNUP50 | GCAAAGGTTCTCTTGCTGC |
| anti-miR-197-3p | miR20000227-1-5, RiboBio,China |
| miR-197-3p mimic | miR10000227-1-5, RiboBio,China |

**Additional file 1: Table S3. The sequence of primers**

| circNUP50-F | TGACAGTGGTGAATGCAAAGG |
| --- | --- |
| circNUP50-R | TACTGGAAGCAAGGCCAGAG |
| BIRC3-F | GCTTTTGCTGTGATGGTGGACTC |
| BIRC3-R | CTTGACGGATGAACTCCTGTCC |
| BCL2-F | ATCGCCCTGTGGATGACTGAGT |
| BCL2-R | GCCAGGAGAAATCAAACAGAGGC |
| UBE2T-F | AGCTGCTCATGTCAGAACCCAAC |
| UBE2T-R | GTCTGGCATTCTTGAGGAAGGC |
| G3BP1-F | AGCCTGTTCAGAAAGTCCTTAGC |
| G3BP1-R | CGAAGGCGATTATCTCGTCGGT |
| miR-1296-5p-F | CgTTAgggCCCTggCTCC |
| miR-197-3p-F | gCgTTCACCACCTTCTCCA |
| U6-F | ATTGGAACGATACAGAGAAGATT |
| U6-R | GGAACGCTTCACGAATTTG |
| GAPDH-F | GTCTCCTCTGACTTCAACAGCG |
| GAPDH-R | ACCACCCTGTTGCTGTAGCCAA |
