# Supplementary figures and images for: CircNUP50 is a novel therapeutic target that promotes cisplatin resistance in ovarian cancer by modulating p53 ubiquitination
Source: J Nanobiotechnology. 2024 Jan 19;22:35. doi: 10.1186/s12951-024-02295-w (PMC10799427; doi:10.1186/s12951-024-02295-w)

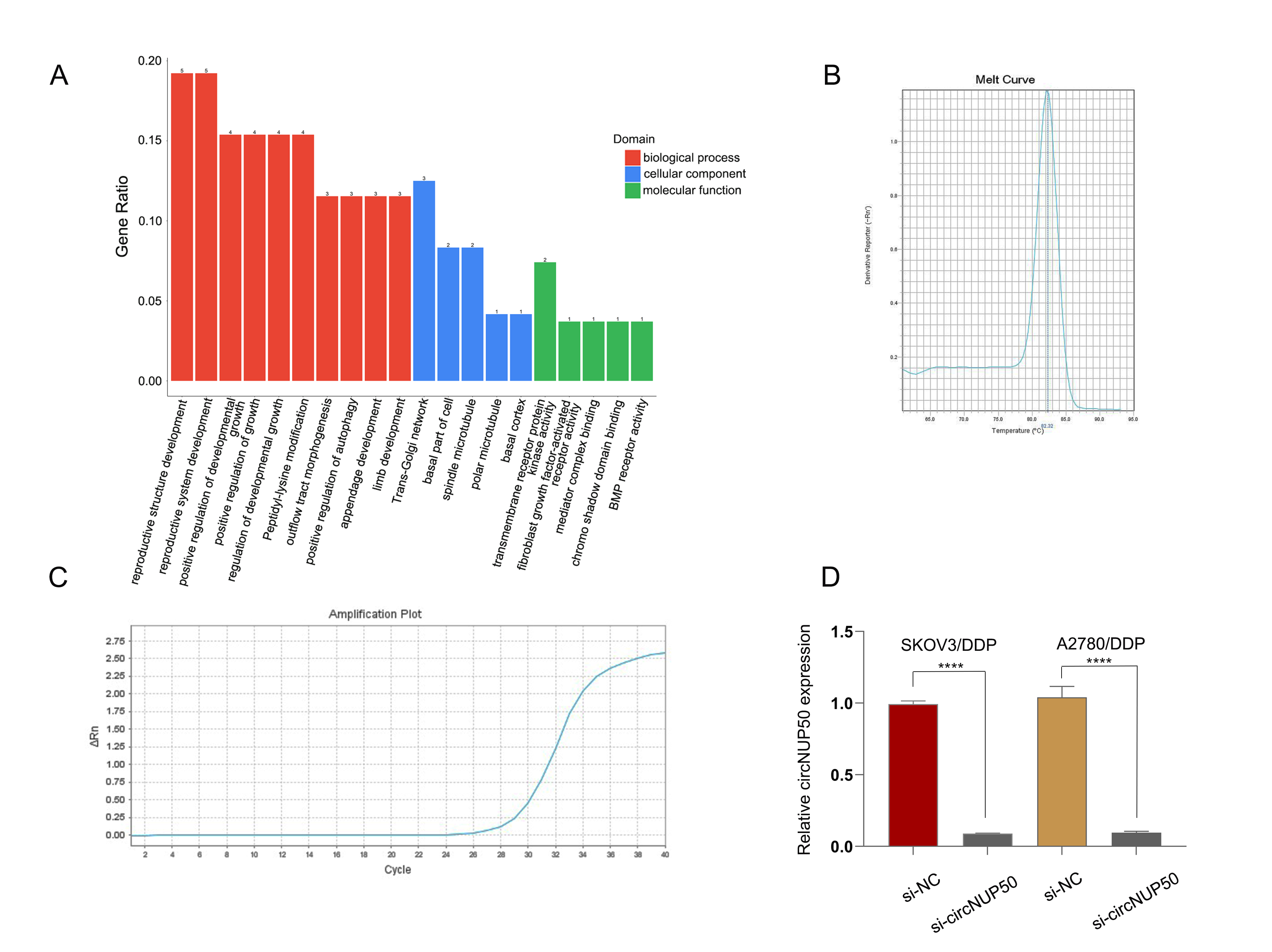

Supplement: Supplementary file 4 — Additional file 4: Figure S1: (A) Gene Ontology (GO) analysis based on circular RNA-sequencing (circRNA-seq) data. (B) and (C) Melt and separation curves display the primers used for circNUP50 amplification in qRT-PCR experiments. (D) qRT-PCR showing that si-circNUP50 could significantly inhibit circNUP50 expression in OC cells. [file 12951_2024_2295_MOESM4_ESM.tif]

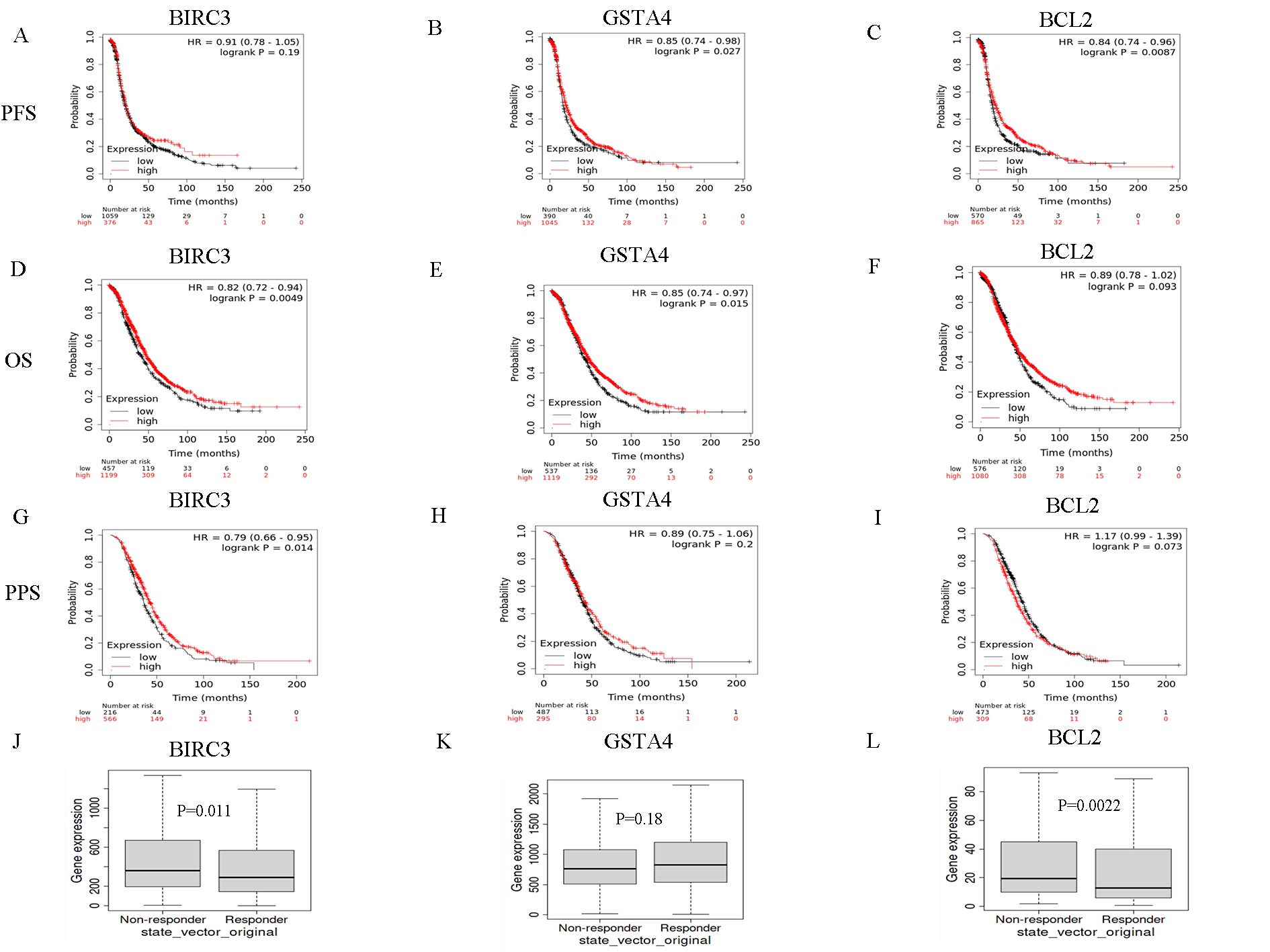

Supplement: Supplementary file 5 — Additional file 5: Figure S2: (A), (D), (G) Association of BIRC3 expression with progression-free survival (PFS), overall survival (OS), and PPS in patients with OC. (B), (E), (H) Association of GSTA4 expression with PFS, OS and PPS in patients with OC. (C), (F), (I) Association of BCL2 expression with PFS, OS and PPS in patients with OC. (J), (K), (L) Correlation of the three gene expressions (BIRC3, GSTA4, and BCL2) with the efficacy of platinum-containing therapy. [file 12951_2024_2295_MOESM5_ESM.jpg]

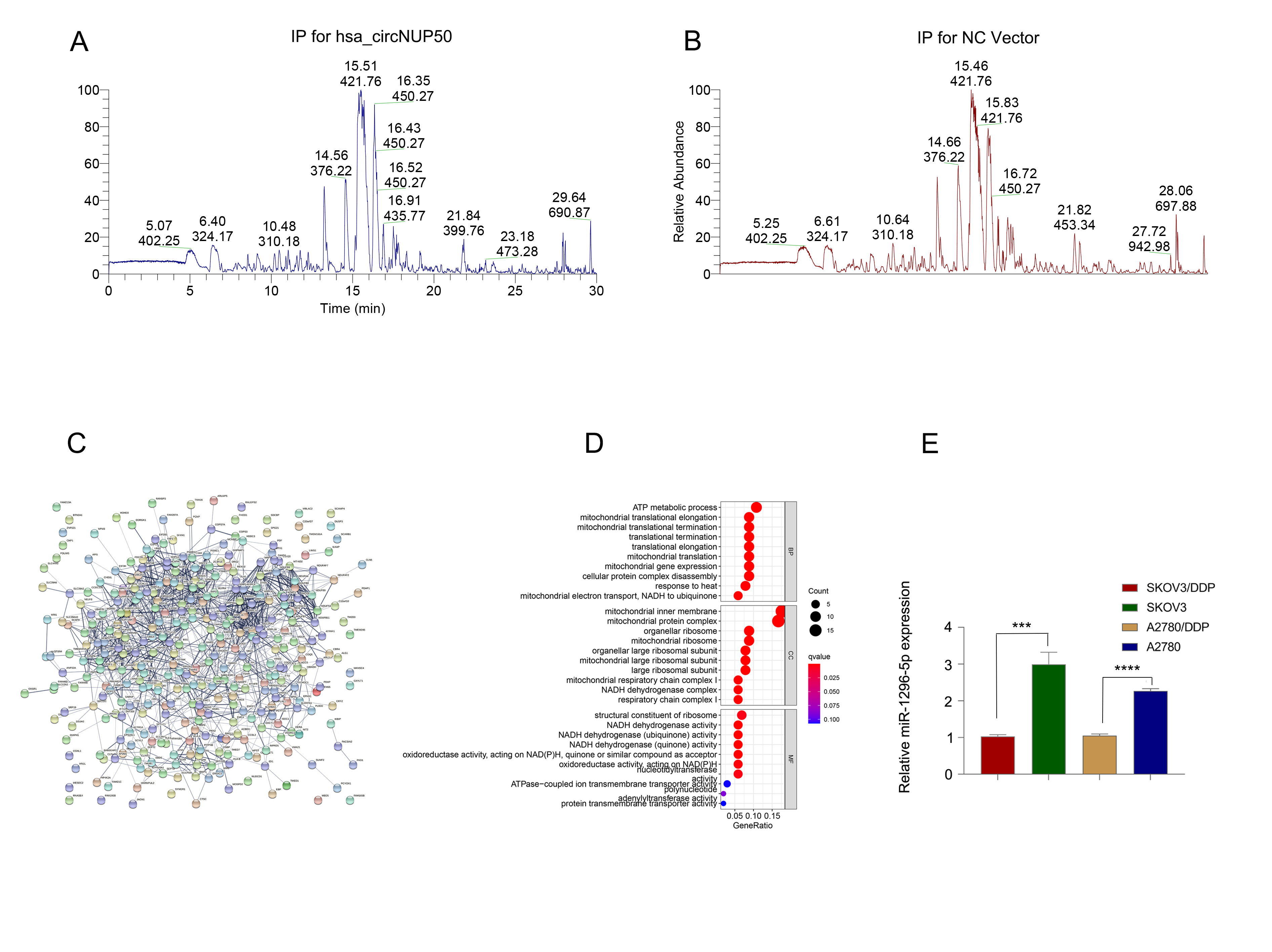

Supplement: Supplementary file 6 — Additional file 6: Figure S3: (A) and (B) Mass spectrometry results of circNUP50-binding proteins. (C) PPI analysis based on mass spectrometry data of circNUP50-binding proteins. (D) GO analysis based on mass spectrometry data. (E) The expression of miR-1296-5p in platinum-resistant and platinum-sensitive (PS) cells was not as significant as that of miR-197-3p. [file 12951_2024_2295_MOESM6_ESM.tif]

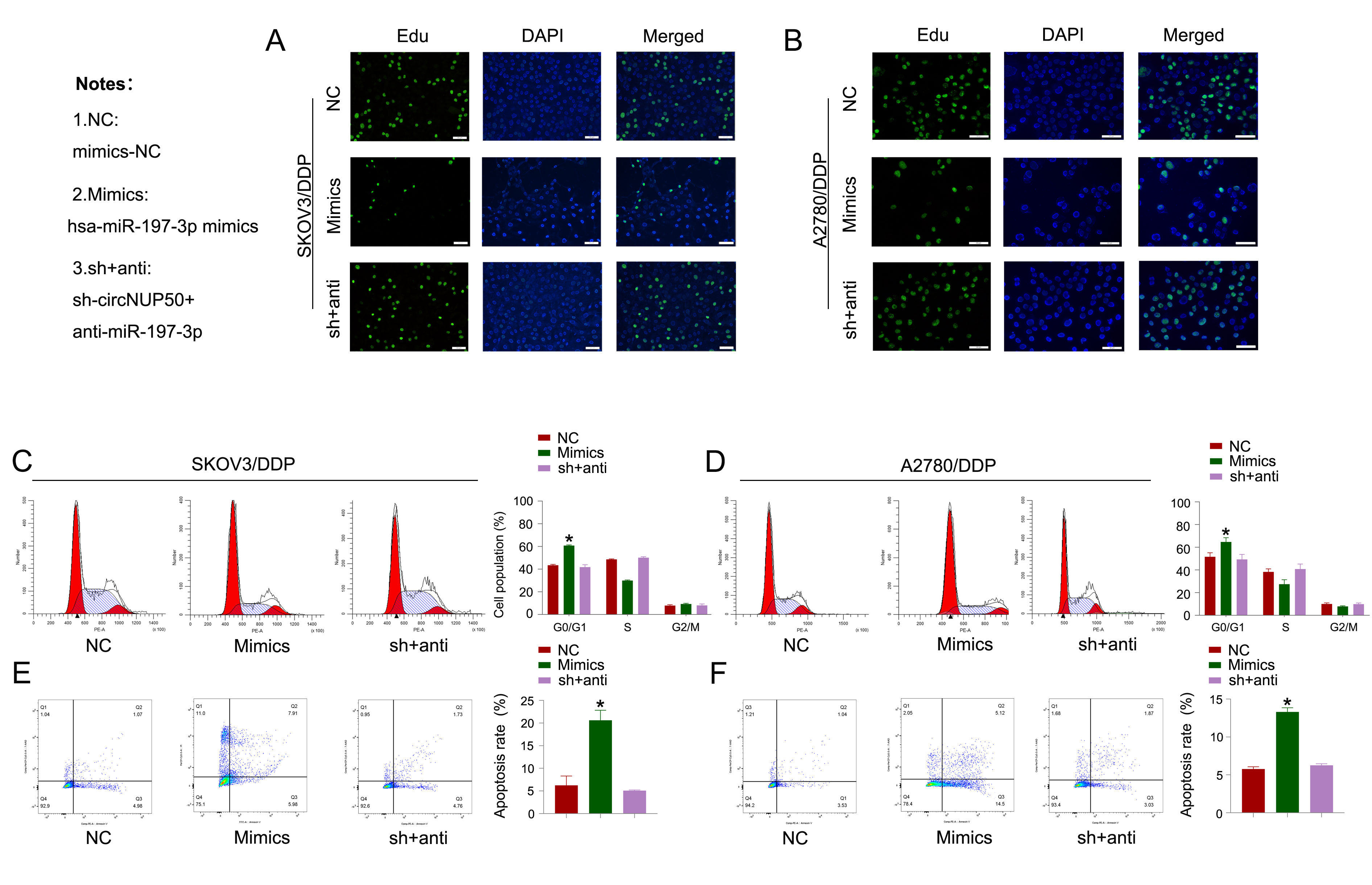

Supplement: Supplementary file 7 — Additional file 7: Figure S4: (A) and (B) 5-ethynyl-20-deoxyuridine (EdU) experiments showed that the proliferation of SKOV3/DDP and A2780/DDP cells reduced when miR-197-3p was overexpressed. (C) and (D) Cell cycle analysis showed that SKOV3/DDP and A2780/DDP cells were arrested in the G0/G1 phase of the cell cycle when miR-197-3p was overexpressed. (E) and (F) Apoptosis experiments showed that the apoptosis rate of SKOV3/DDP and A2780/DDP cells was increased after miR-197-3p overexpression. [file 12951_2024_2295_MOESM7_ESM.tif]

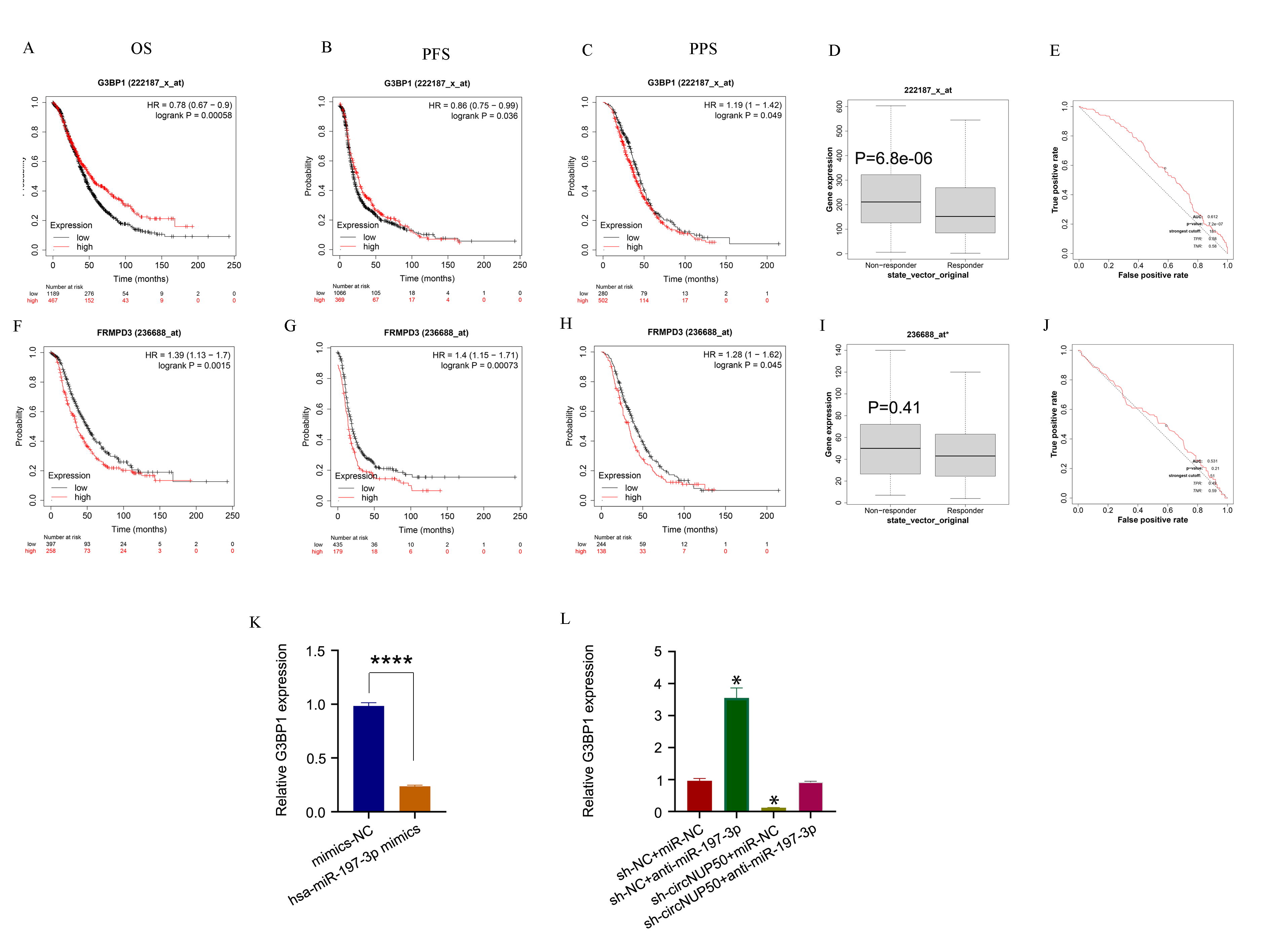

Supplement: Supplementary file 8 — Additional file 8: Figure S5: (A–C) Association of G3BP1 expression with OS, PFS and PPS in patients OC. (D) and (E) G3BP1 expression significantly differed from patients who responded to platinum-containing therapy. (F–H) Association of FRMPD3 expression with OS, PFS and PPS in patients with OC. (I) and (J) FRMPD3 expression was not significantly different in patients who responded to platinum-containing therapy. (K) qRT-PCR showed that G3BP1 expression was significantly decreased when miR-197-3p mimics were overexpressed. (L) In contrast to sh-circNUP50 transfection, anti-miR-197-3p transfection in SKOV3/DDP cells resulted in elevated G3BP1 expression. [file 12951_2024_2295_MOESM8_ESM.tif]

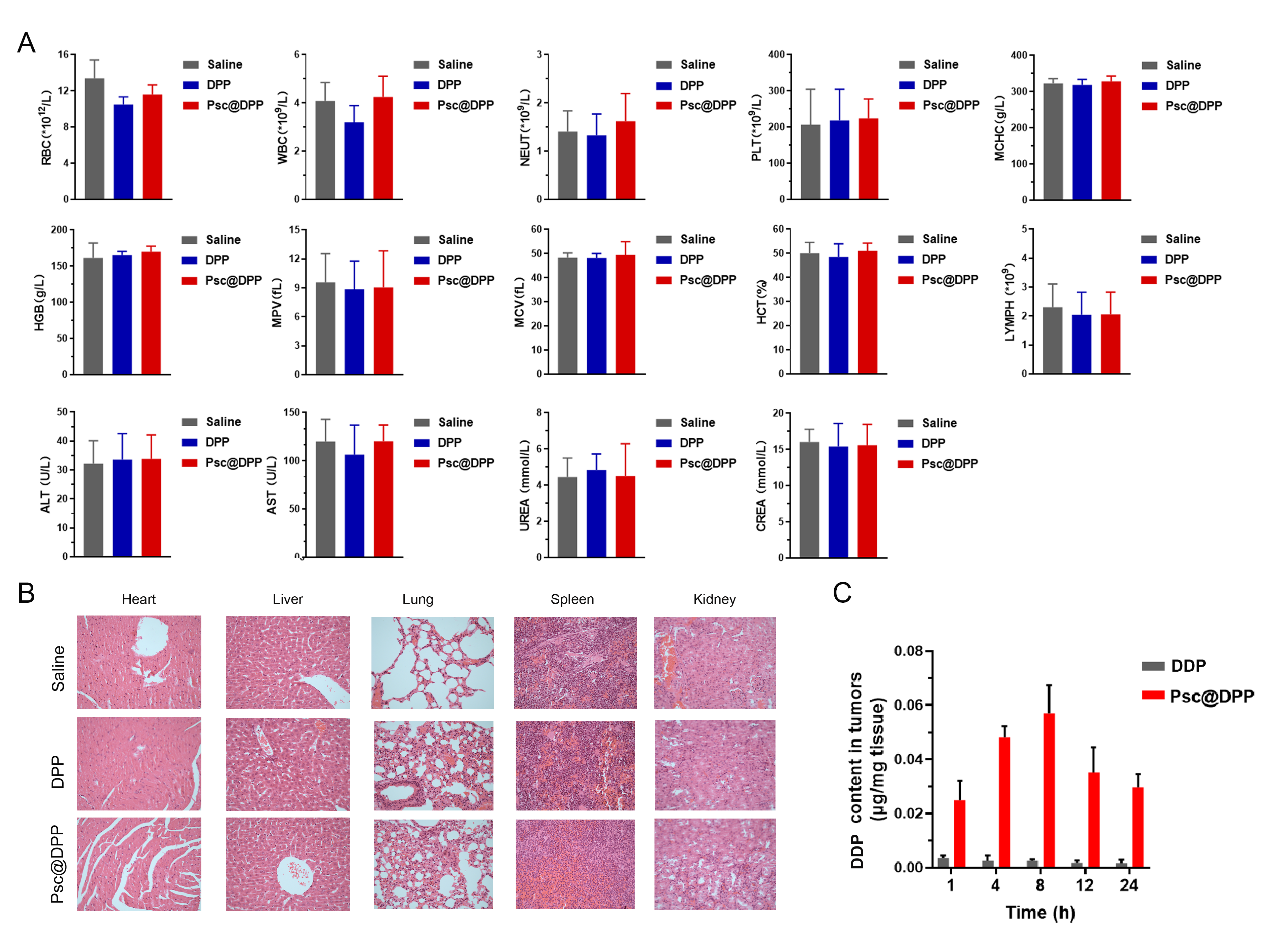

Supplement: Supplementary file 9 — Additional file 9: Figure S6: Safety (A), organ damage (B) and tumour targeting (C) properties of the Psc@DPP nanosystem. [file 12951_2024_2295_MOESM9_ESM.tif]

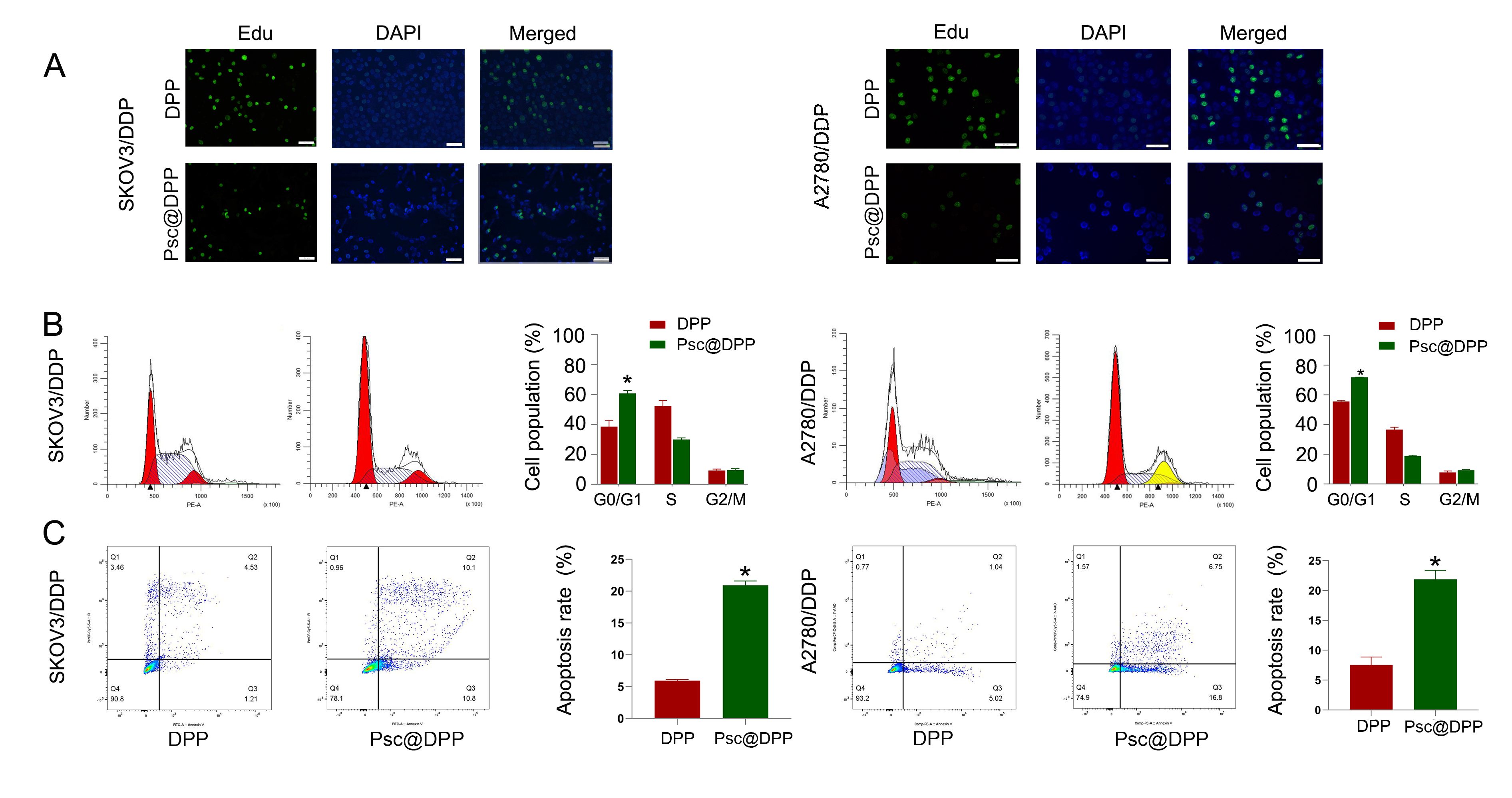

Supplement: Supplementary file 10 — Additional file 10: Figure S7: Potential anti-tumour effects of the Psc@DPP nanosystem on cell proliferation (A), cell cycle (B), and cell apoptosis (C). [file 12951_2024_2295_MOESM10_ESM.tif]
